# Lignans: A Chemometric Analysis

**DOI:** 10.3390/molecules23071666

**Published:** 2018-07-09

**Authors:** Lisa I. Pilkington

**Affiliations:** School of Chemical Sciences, The University of Auckland, Private Bag 92019, Auckland 1142, New Zealand; lisa.pilkington@auckland.ac.nz; Tel.: +64-9-373-7599 (ext. 86776)

**Keywords:** lignans, chemometrics, neolignans, flavonolignans, chemical space, drug-like

## Abstract

The physicochemical properties of classical lignans, neolignans, flavonolignans and carbohydrate-lignan conjugates (CLCs) were analysed to assess their ADMET profiles and establish if these compounds are *lead-like*/*drug-like* and thus have potential to be or act as leads in the development of future therapeutics. It was found that while no studied compounds were *lead-like,* a very large proportion (>75%) fulfilled all the requirements to be deemed as present in *drug-like* space and almost all compounds studied were in the known drug space. Principal component analysis was an effective technique that enabled the investigation of the relationship between the studied molecular descriptors and was able to separate the lignans from their sugar derivatives and flavonolignans, primarily according to the parameters that are considered when defining chemical space (i.e., number of hydrogen bond donors, acceptors, rotatable bonds, polar surface area and molecular weight). These results indicate that while CLCs and flavonolignans are less *drug-like,* lignans show a particularly high level of *drug-likeness*, an observation that coupled with their potent biological activities, demands future pursuit into their potential for use as therapeutics.

## 1. Introduction

Lignans are a class of secondary metabolites that are derived from the oxidative dimerisation of two or more phenylpropanoid units [1]. Despite their common biosynthetic precursors, lignans show vast structural diversity due to the numerous potential coupling modes of the phenoxy radicals [2]. The nature of the molecular linkage of the phenylpropanoids provides the most fundamental level of classification of lignans into two main subclasses—classical lignans and neolignans—although there exist other smaller subclasses, including flavonolignans and coumarolignans [1,3,4,5,6]. 

**Figure 1:**
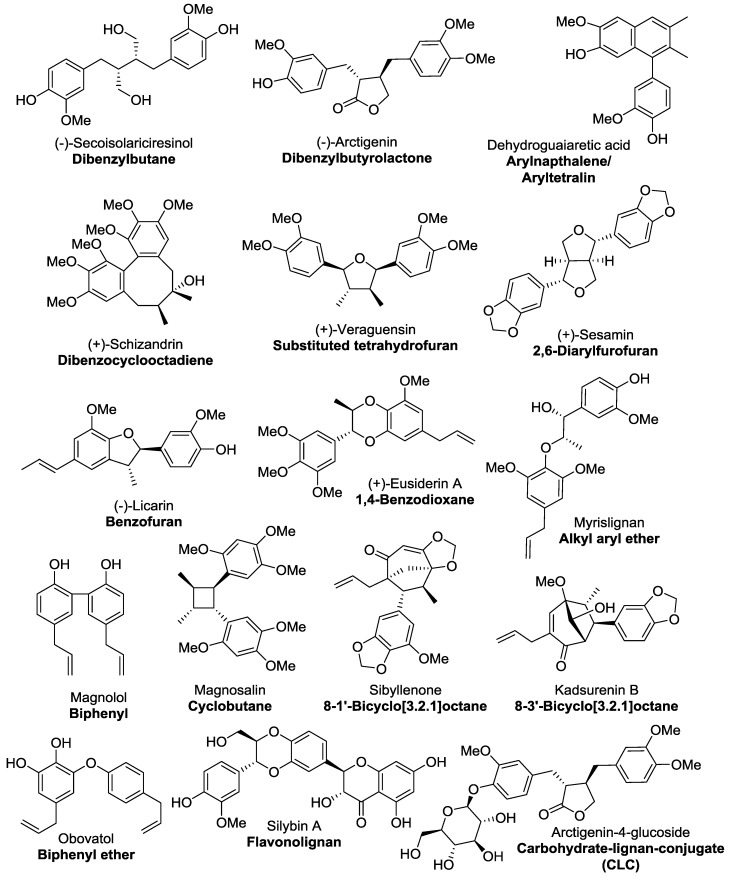
Examples of the six types of classical lignans, the eight types of neolignans investigated in this study, a flavonolignan and carbohydrate-lignan conjugate (CLC).

Classical lignans are phenylpropane dimers that have a β-β′ linkage and there six main subtypes of classical lignans—dibenzylbutanes, dibenzylbutyrolactones, arylnaphthalenes/aryltetralins, dibenzocyclooctadienes, substituted tetrahydrofurans, and 2,6-diarylfurofurans (Figure 1) [3,6,7]. Neolignan was a classification initially coined by Gottlieb to distinguish phenylpropanoid dimers that did not contain the β-β′ (also referred to as an 8-8′) phenylpropane linkage characteristic of classical lignans [8,9]. Neolignans have more varied structures than classical lignans; there are 15 subtypes designated by the nature and position of the linkage between the phenylpropane units [3,6,10], the most common subtypes being benzofurans, 1,4-benzodioxanes, alkyl aryl ethers, biphenyls, cyclobutanes, 8-1′-bicyclo[3.2.1]octanes, 8-3′-bicyclo[3.2.1]octanes and biphenyl ethers, examples of which are shown in Figure 1. 

Lignans have been found in more than 70 plant families and an extensive range of localities within plants, from roots to leaves, seeds and flowers [1,3,4,5,11]. Most importantly, this class of compound has exhibited several potent, significant, biological activities, including anticancer [3,4,12,13], antimicrobial [4], antiviral [12,13,14,15], immunosuppressive [4], anti-inflammatory [4], antioxidant [3,4,16], and hepaprotective [15,16,17] actions as well as cancer [18,19] and osteoporosis [20] prevention properties; activities that have contributed an ever-increasing interest in lignans and their synthesis [3,4,5,7,11,15,21,22,23,24,25,26,27,28,29,30,31,32,33,34,35,36,37,38,39,40,41].

Throughout human history, plants with a high lignan content have been utilised to treat illnesses and ailments, playing a vital role in traditional folk medicine [5,11]. These lignan-containing plants have been documented in medical pharmacopoeias from a large number of cultures including English, Korean, Native American, Chinese, Japanese, South American and Tibetan. For many, the uptake of modern medicine has supplanted the need and use of traditional medicines, however the continued use of folk medicine exists in a large number of cultures including Ayurvedic, Unani, Siddhi, Kampo, Jamu and in traditional Chinese medicine [4]. Furthermore, traditional medicine has been a critical source of inspiration in the pursuit of modern drug therapies, with a number of presently-used medicines being engendered by compounds of natural origins–circa 40% of commercially-available drugs are either natural products or derivatives thereof [5]. In this capacity, lignans constitute an important class of compounds that provide a starting point for the development of therapeutic agents [1,3,5,11,21,42,43,44].

**Figure 2:**
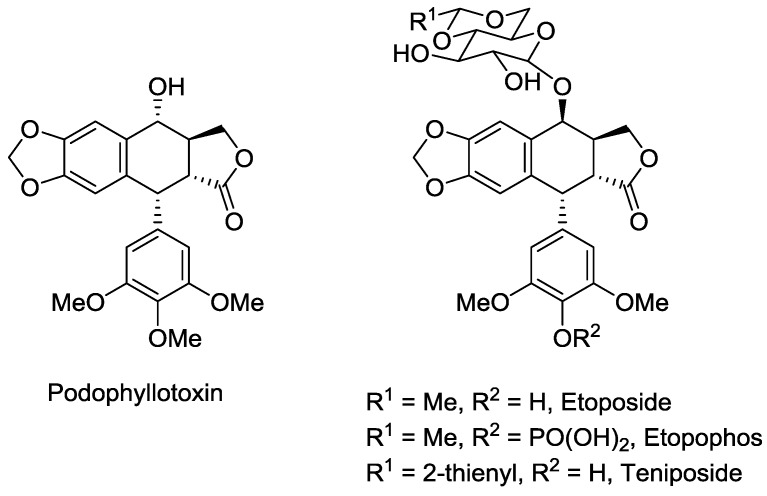
Structure of podophyllotoxin and three of its most notable derivatives; etoposide, etopophos and teniposide.

Potentially the most well-known example of a lignan as a currently-utilised and lead compound is the aryltetralin lactone, podophyllotoxin (Figure 2) [11,21,45]. It has been known for centuries that the plants of the *Podophyllum* genus possess medicinal properties. These plants have particularly been used by the indigenous peoples of the Himalayas and North America [45]. Podophyllotoxin was first isolated in 1880 from one of these plants [46], and is a cytotoxic compound that binds to tubulin, thereby inhibiting microtubule assembly during mitosis and thus interrupts the cell cycle [47,48,49]. Podophyllotoxin has a mode of action and level of potency that lends it to be a possible cancer chemotherapeutic, however this possibility was tempered by the discovery that it exhibits high levels of gastrointestinal toxicity [50]. Podophyllotoxin, however, has been approved for use as a topically-administered treatment for genital warts. Additionally, podophyllotoxin was used as a lead compound for antitumour agents [51], resulting in the development of etoposide, its water-soluble phosphate ester prodrug, etopophos and teniposide as anticancer agents that are all in current use to treat a range of cancers, including testicular, lung and ovarian cancer, lymphoma, leukemia, neuroblastoma and various types of brain tumours [45,50,52]. It should be noted that etoposide, etopophos and teniposide exhibit an alternative mode of action to their lead compound, podophyllotoxin, in that they are potent DNA topoisomerase II inhibitors [50,51,52].

Podophyllotoxin provides an inspiring example of the potential that lignans possess as a foundation for the development of medicines to target diseases and conditions, many of which that have an unmet need for cures and treatments. 

While potent biological activity, which many lignans possess, is the most critical property of a potential drug or lead compound, it is also important to assess the Absorption, Distribution, Metabolism, Excretion and Toxicity (ADMET) profiles of these compounds to evaluate their likelihood of being effective drug leads [53]. To do this, the physicochemical properties of the compounds can be calculated–these molecular descriptors can subsequently be assessed against various existing and verified benchmarks. *Drug-like* chemical space is defined by the Lipinski’s rule of five–the most widely used and recognised set of parameters that are used to assess properties of potential therapeutics. Compounds that fall within these boundaries are indicated to be able to be orally absorbed [54,55]. The two other definitions of chemical space are *lead-like* space and known drug space (KDS). Compounds within *lead-like* chemical space are typically compounds that are less complex, hence have low molecular weights and lower lipophilicities (LogP) [56]—*lead-like* compounds have very low limits for these parameters as they generally increase during the optimisation process in medicinal chemistry; *lead-like* compounds are more likely to become real therapeutics once modified [57,58]. KDS is defined by criterion that includes all small organic compounds that have been assessed in human clinical trials and were/are subsequently in medical use [59]. The upper limits for each chemical space referred to in this study are provided (Table 1). 

We wished to assess the ADMET profile of lignans and related compounds to explore their position in the predefined chemical spaces, as well as if there are notable differences within, and between, these groups. Presented herein is the result of the investigation into the physicochemical properties of traditional lignans, neolignans, flavonolignans and sugar derivatives of lignans (carbohydrate-lignan conjugates; CLCs) to establish if these compounds are *lead-like*/*drug-like* and thus have potential to be or act as leads in the development of future therapeutics. 

## 2. Methodology

Representative compounds for each of the main subclasses of lignan and neolignan compounds were found by doing a substructure search using Scifinder and choosing the ten lignan compounds with the highest number of references. There were six subclasses of classical lignans (dibenzylbutanes, dibenzylbutyrolactones, arylnapthalenes/aryltetralins, dibenzocyclooctadienes, substituted tetrahydrofurans and 2,3-diarylfurans) and eight subclasses of neolignans (benzofurans, 1,4-benzodioxanes, alkyl aryl ethers, biphenyls, cyclobutanes, 8-1′-bicyclo[3.2.1]octanes, 8-3′-bicyclo[3.2.1]octanes and biphenyl ethers) included in this study–examples of these subclasses are given in Figure 1 and the compound details (name, class and CAS number) of each compound included in this study are given in the Supplementary Information. Furthermore, additional lignan-like compounds were also found–flavonolignans and sugar derivatives (carbohydrate-lignan conjugates, CLCs) of classical lignan and neolignan subclasses. In total, 16 different groups of compounds were studied, each group consisting of ten compounds. Hence, 160 compounds were included in this representative study. 

The 3D structures of the compounds were drawn using ChemBioDraw as part of the ChemOffice software package [60]. The structures were then optimised using the MM2 [61] force field in Chem3D [60]. The molecular descriptors were calculated using QikProp 4.42 [62], which has been shown to be an accurate and reliable tool for the calculation of the molecular descriptors analysed in this study [63]. 

Following generation of the molecular descriptors for all the compounds in the study, the mean, median and standard deviation of each descriptor was calculated (see Section 3.1). Graphs of the distributions of these molecular descriptors were generated with R (version 3.2.2) [64] and R Studio (version 0.99.486) [65] using the ggplot2 package [66]. Compounds were categorised as *lead-like*, *drug-like* and in KDS for each of the parameters by comparing the values for the descriptors against those stated in Table 1 and including them in the chemical space if the calculated value was less than or equal to the stipulated benchmark.

Principal Component Analysis (PCA) was carried out using all compounds and parameters included in this study (see Section 3.2) using R (version 3.2.2) [64] and R Studio (version 0.99.486) [65]. PCA analysis was performed using the prcomp function as part of the stats package, by singular value decomposition of the centred and scaled data matrix [64]. Results of this analysis were visualised using the factoextra package (version 1.0.5) [67]. 

## 3. Results and Discussion

### 3.1. Molecular Descriptors

Using the aforementioned methods, ten molecular descriptors were calculated for each of the 160 compounds studied. Molecular weight, lipophilicty (LogP), the number of hydrogen bond donors, hydrogen bond acceptors and rotatable bonds and polar surface area (PSA) have been extensively used in the assessment of a molecules’ suitability to be considered as a drug [68]. The other molecular descriptors—dipole moment, polarisability, ionisation potential and water solubility (LogS) have been used less extensively, however their association with desirable characteristics has led them to being increasingly examined in recent times [63,69,70].

To analyse the molecular descriptors, summary statistics–the mean, median and standard deviation for each of these parameters—for each compound type, as well as for all 160 compounds (all classical lignans, neolignans, flavonolignans and CLCs) were calculated and are in the table provided (Table 2).

#### 3.1.1. Molecular Weight

**Figure 3:**
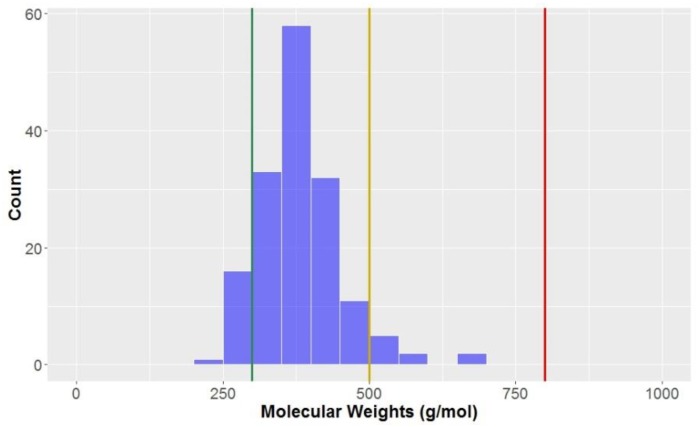
The statistical distribution of the molecular weight of all analysed compounds (green = 300 g mol^−1^, compounds < 300 g mol^−1^ are in the *lead-like* space; yellow = 500 g mol^−1^, compounds < 500 g mol^−1^ are in the *drug-like* space; red = 800 g mol^−1^, compounds < 800 g mol^−1^ are in the KDS). Total number of compounds = 160.

The molecular weights of the compounds in this study are approximately normally distributed (Figure 3), with an overall mean of 381.5 g mol^−1^ and standard deviation of 70.4 g mol^−1^ (Table 2). 

Unsurprisingly, the categories with the highest average molecular weights were CLCs and flavonolignans, with mean molecular weights of 567.4 ± 67.5 and 478.6 ± 7.0 g mol^−1^, respectively. By definition, flavonolignans are the result of a dimerisation of a phenyl propanoid unit and flavone nucleus, a flavone moiety having a higher molecular weight than another phenyl propanoid unit that forms the basis of a classical lignan/neolignan. The CLCs in this study are classical lignans/neolignans with a least one additional saccharide unit attached. Of all of the sub-classes, flavonolignans had the lowest standard deviation for molecular weight, indicative that the compounds of this type have very similar molecular composition. Of the classical lignans and neolignans, dibenzyocyclooctadienes had a significantly higher average than other classical lignans and neolignans (413.3 g mol^−1^ vs. 361.2 g mol^−1^). Conversely, biphenyls (313.8 ± 62.6 g mol^−1^) and biphenyl ethers (296.5 ± 27.4 g mol^−1^) had the lowest molecular weights, on average. Looking at these compounds, they generally have lower numbers of substituents on the aromatic ring and less elaboration of the sidechains, which could account for this observation. Compounds in the KDS have molecular weights lower than 800 g mol^−1^ (red line in Figure 3); as can be seen, all of the compounds studied exist in KDS for this parameter. Almost all (94.5%) of the compounds would be considered to be *drug-like* when considering molecular weight, however only ~10% of compounds are also considered *lead-like* (<300 g mol^−1^). 

#### 3.1.2. The Octanol–Water Partition Coefficient (LogP)

**Figure 4:**
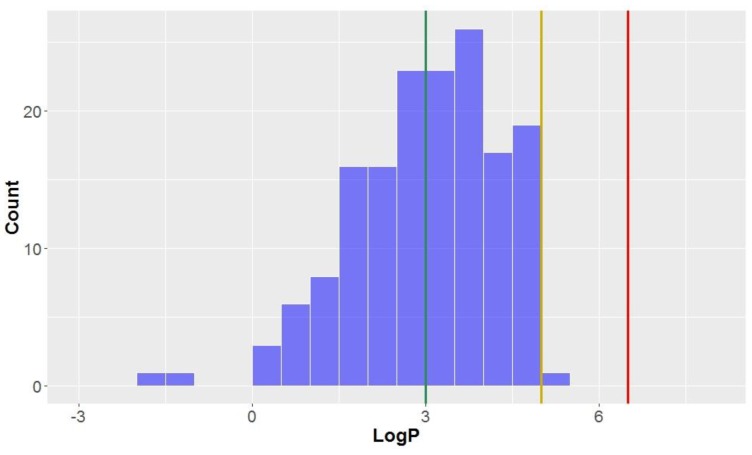
The statistical distribution of the octanol–water partition coefficient (LogP) of all analysed compounds (green = 3, compounds < 3 are in the *lead-like* space; yellow = 5, compounds < 5 are in the *drug-like* space; red = 6.5, compounds < 6.5 are in the KDS). Total number of compounds = 160.

Like the molecular weights, the lipophilicites (LogP values)—the octanol-water partition coefficient of the molecules—are approximately normally distributed (mean = 3.0, standard deviation = 1.3, Table 2, Figure 4). All compounds studied have a calculated LogP less than the benchmark for KDS (LogP = 6.5), and all but one can be considered *drug-like* (LogP < 5) for this parameter. Approximately half of the compounds had a calculated lipophilicity allowing it to be in *lead-like* space. The compound classes that were calculated to exhibit the highest degree of lipophilicity were dibenzocyclooctadienes and cyclobutanes (LogP = 3.9 ± 0.7 and 3.7 ± 0.9, respectively). Contrastingly, CLCs have the lowest average calculated LogP (0.4 ± 1.1), thereby demonstrating a low affinity for non-aqueous systems and the highest degree of hydrophilicity. Flavonolignans also had low LogP values (mean = 1.6) which was notably lower than the classical lignans and neolignans studied (mean = 3.3). 

#### 3.1.3. Hydrogen Bond Donors and Acceptors

**Figure 5:**
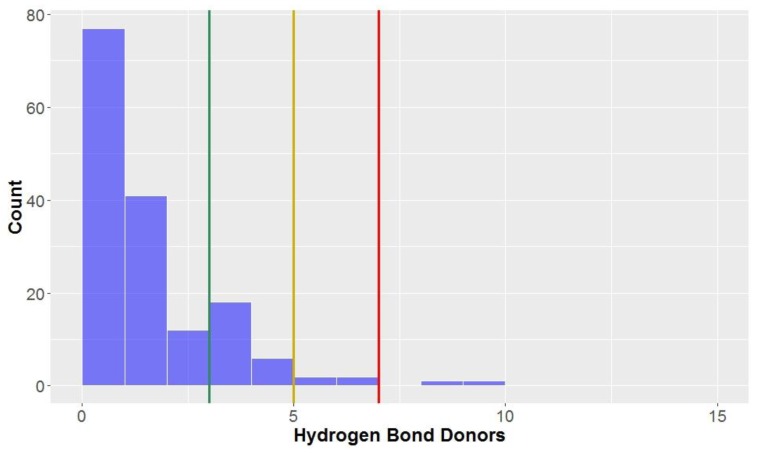
The statistical distribution of the hydrogen bond donors of all analysed compounds (green = 3, compounds < 3 are in the *lead-like* space; yellow = 5, compounds < 5 are in the *drug-like* space; red = 7, compounds < 7 are in the KDS). Total number of compounds = 160.

Ideally, compounds should not have too many hydrogen bond donors and acceptors; the number of hydrogen bond donors should be lower than seven, five and three to be considered to be in KDS, *drug-like* space and *lead-like* space, respectively. On average, the compounds in this study conform reasonably well with the three aforementioned definitions used for the chemical spaces (mean = 3.0, standard deviation = 1.3, Figure 5, Table 2) for hydrogen bond donors. As can be seen, most compounds have three or less hydrogen bond donors (81.3%), allowing them to be classified in *lead-like* space and the majority of compounds have less than two. There are a proportion of compounds that do have more than three hydrogen bond donors–these compounds were mainly CLCs and flavonolignans, with their mean number of hydrogen bond donors being 6.1 and 4.1, respectively. Dibenzylbutanes and alkyl aryl ethers also had a significant percentage of compounds excluded from *lead-like* space according to this parameter. 

Only 15% of compounds were classified as being *lead-like* in terms of the number of hydrogen bond acceptors (≤3 hydrogen bond acceptors)–much lower than that observed for the hydrogen bond donors, although greater than 90% of compounds had ≤10 hydrogen bond acceptors, classifying them as *drug-like.*

**Figure 6:**
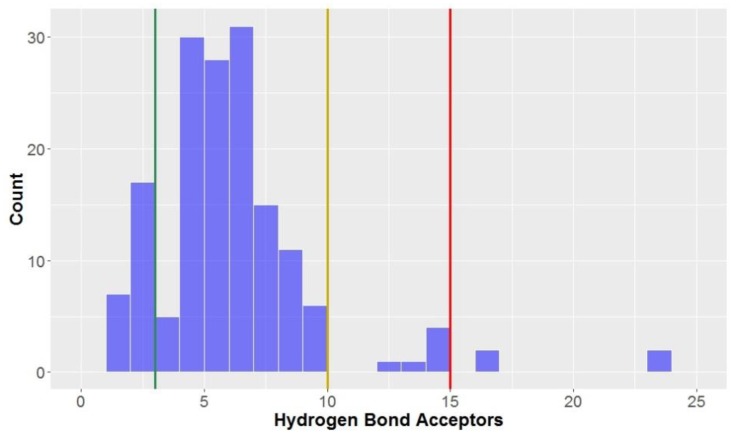
The statistical distribution of the hydrogen bond acceptors of all analysed compounds (green = 3, compounds < 3 are in the *lead-like* space; yellow = 5, compounds < 5 are in the *drug-like* space; red = 15, compounds < 15 are in the KDS). Total number of compounds = 160.

The number of hydrogen bond acceptors displayed a slightly-left skewed normal distribution (Figure 6)–far different to the strongly-skewed distribution seen for the aforementioned number of hydrogen bond donors (Figure 5). The overall mean number of hydrogen bond acceptors was 6.3, although this was largely inflated due to the CLCs (hydrogen bond donors = 16.6 ± 3.8) and to a lesser degree, flavonolignans (hydrogen bond donors = 9.0 ± 0.8); without these two compound types included in the analysis, the mean decreased to 5.4 hydrogen bond acceptors. The only compounds studied with greater than 15 hydrogen bond acceptors, thus not in KDS, were CLCs. 

#### 3.1.4. Polar Surface Area (PSA)

**Figure 7:**
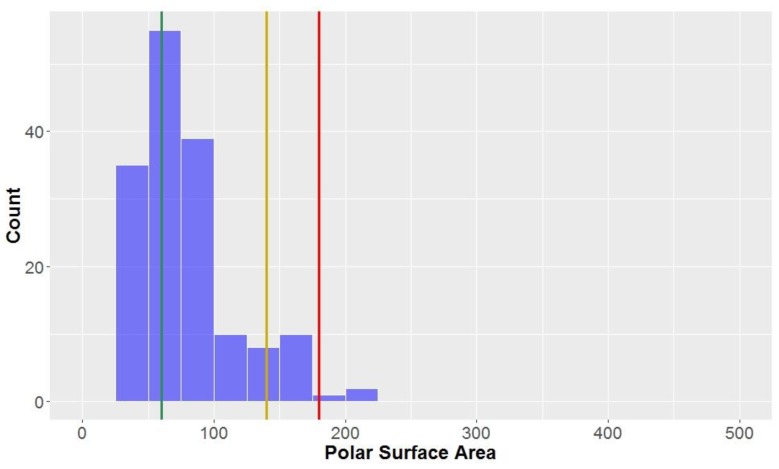
The statistical distribution of the polar surface area (PSA) of all analysed compounds (green = 60, compounds < 60 Å^2^ are in the *lead-like* space; yellow = 140, compounds < 140 Å^2^ are in the *drug-like* space; red = 180, compounds < 180 Å^2^ are in the KDS). Total number of compounds = 160.

The polar surface area (PSA) of all the compounds in the study was found to be 79.5 ± 37.1 Å^2^ (Figure 7, Table 2). This parameter is inherently-linked to the number of hydrogen bond acceptors and donors, thus it is no surprise that CLCs had a much higher average polar surface area than the overall average (PSA = 156.9 ± 29.3 Å^2^). CLCs, however, did not have the highest mean PSA–flavonolignans had a marginally higher average PSA (158.5 ± 11.4 Å^2^). Of the classical lignans/neolignans, the subclass with the highest PSA were dibenzylbutyrolactones (PSA = 90.8 ± 13.6 Å^2^); the compounds with the lowest PSAs were dibenzylcyclooctadienes and substituted THF’s (mean = 51.2 Å^2^ and 50.6 Å^2^, respectively). The highest PSA at which oral absorption is able to occur has been reported to be 140 Å^2^ – this value thereby benchmarks the upper PSA limit for *drug-like* space [71,72]. A large proportion of studied compounds (88.1%) are in *drug-like* space when considering PSA, while nearly all compounds are in KDS (PSA ≤ 180 Å^2^), while only a third of compounds were within the strict bounds of *lead-like* space.

#### 3.1.5. Rotatable Bonds

**Figure 8:**
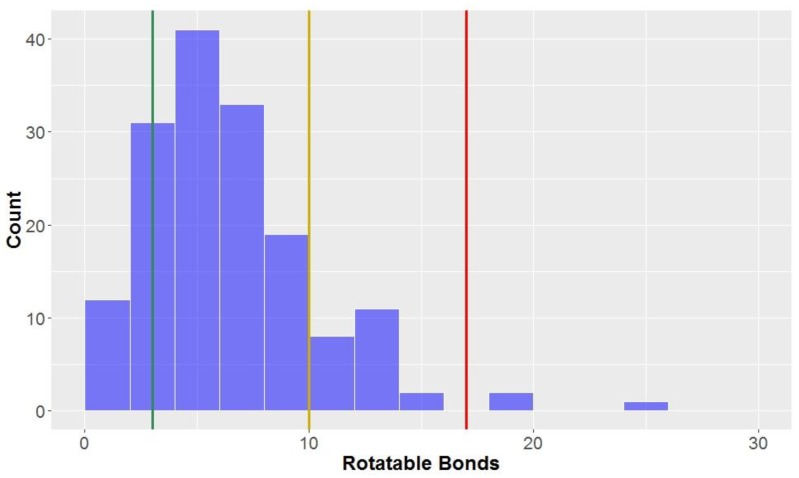
The statistical distribution of the rotatable bonds of all analysed compounds (green = 3, compounds < 3 are in the lead-like space; yellow = 10, compounds < 10 are in the drug-like space; red = 17, compounds < 17 are in the known drug space). Total number of compounds = 160.

Looking at the general structures of classical lignan/neolignan types, it is apparent that most, in addition to the aryl rings present, have additional ring cycles present in the structures (Figure 1). The exceptions to this general rule are the biphenyl structures, dibenzylbutanes and alkyl aryl ethers; this is reflected in the number of rotatable bonds (Table 2, Figure 8), where alkyl aryl ethers and dibenzylbutanes have the largest average number of rotatable bonds of all the classical lignans/neolignans (mean = 12.1 and 11.0, respectively). CLCs also had high counts for number of rotatable bonds, whereas highly-constricted structures, with a more fused-ring scaffold had far lower averages for this parameter, i.e., 3.3 ± 2.2 for 2,6-diarylfurofurans, and mean = 3.4 ± 1.6 for substituted tetrahydrofurans and arylnapthalenes/aryltetralins. 

The number of rotatable bonds had the second-lowest proportion of compounds classified as *lead-like*, (12.5%) indicating that this parameter (along with molecular weight with 10.6% in *lead-like* space) is one of the more effective descriptors for eliminating potential drug candidates. Lu et al. found that compounds were considered to be in *privileged property space* if they had ≤10 rotatable bonds–hence, this is the benchmark used to define *drug-like* space for the number of rotatable bonds [72]. The number of rotatable bonds was the most discerning factor for inclusion of compounds in *drug-like* space (85.0% of compounds met the criteria of ≤10 rotatable bonds, Table 3). However, 98.1% of the compounds tested were within the bounds of KDS, with ≤17 rotatable bonds. 

#### 3.1.6. Other Molecular Descriptors

The calculated dipole moments of the compounds are approximately normally distributed, with a mean of 4.1 ± 1.9 D (Table 2, Figure S1). The compounds with the lowest dipole moments were dibenzocyclooctadienes (mean = 2.6 ± 1.0 D) and 2,6-diarylfurofurans (mean = 2.7 ± 1.3 D), while dibenzylbutyrolactones and biphenyl ethers were the types that had the highest average dipole moments (mean = 5.5 D and 5.3 D, respectively). Density functional theory (DFT) has previously been applied to dipole moment measurements of compounds in KDS, a study which found that compounds within KDS have dipole moments ≤10 [70]. Furthermore, it has been reported that to be orally available, a drug should have a dipole moment <13 D. All the compounds in this study had dipole moments below 10 D, indicating that all of the compounds lie within KDS for this parameter and would be orally-available, using dipole moments as a measure of this desirable characteristic in drug therapeutics.

Intrinsically linked to lipophilicity (LogP), the water solubility (LogS) of a compound is an important property to consider [73]. Akin to the dipole moment, the LogS of a compound can be a signifier of oral-availability; it has been shown that the majority of orally available drugs have a LogS between 0 and −7, centring between −4 and −3 [67]. The distribution of the calculated hydrophilicity of the compounds in this study has an approximately normal distribution in those ranges (Figure S2, Table 2). Using the above-mentioned range as the criterion for this parameter as a yardstick of oral-availability, 97% are likely to be orally-available. The mean LogS was −4.1 ± 1.4 for all compounds and it can be noted that CLCs and dibenzylbutyrolactones (mean = −2.5 and −2.6, respectively) have the highest LogS values and thus greater aqueous solubility. In contrast, cyclobutanes (mean = −5.8 ± 1.5), dibenzylcyclooctadienes (mean = −5.6 ± 0.9), and substituted tetrahydrofurans (mean = −5.4 ± 1.1), had lower mean hydrophilicity values, indicating a low affinity for aqueous media. 

**Figure 9:**
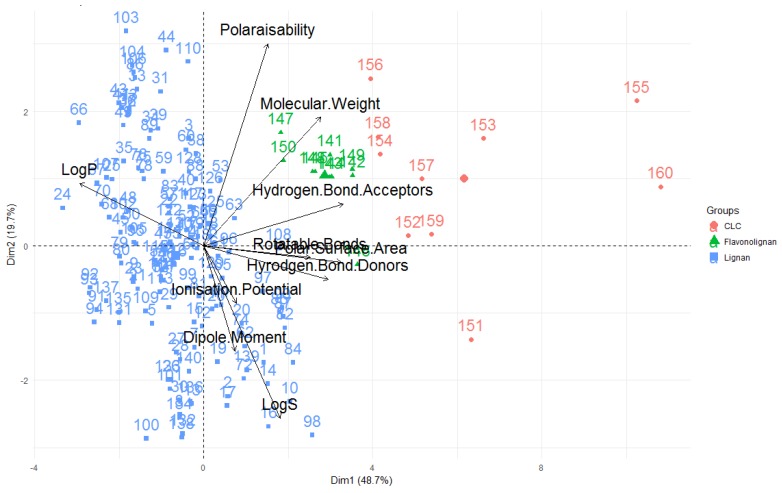
Biplot representing the PCA analysis on the studied compounds and molecular descriptors. The arrows represent molecular descriptors and the direction in which they hold influence. Each point represents a molecule in this study (blue = classical lignans and neolignans, green = flavonolignans, red = CLCs).

The ionisation potential of the compounds were normally distributed but had very low variability (mean = 0.9 and standard deviation = 0.4, for all compounds) and no significant differences between compound groups (Table 2, Figure S3). Analogous to the findings for dipole moment and water solubility, the studied compounds have a high degree of compliance with the benchmarks set for ionisation potentials as an indicator of oral availability. All but two of the 160 compounds investigated had an ionisation potential between 8 and 10 eV–it has been shown that orally administered commercial drugs have ionisation potentials in this range [67]. Of importance to note is that these three descriptors that have been particularly associated with oral availability; ionisation potential, LogS and dipole moment, are very closely correlated in the PCA analysis (see following section for further discussion, Figure 9), with these vectors all having similar bearings. Ionisation potential has also been shown to predicate the redox stability of compounds and thus their ease of metabolism in the body [70].

Polarisability is defined as the ability of a compound to form instantaneous dipoles and can be associated with the ability of the drug to permeate the cell [70]. It has previously been shown that there is a high correlation between the polarisability and molecular weight of a compound (r^2^ = 0.90)–an observation that accounts for why molecular weight is such a crucial parameter in chemical space definition [74]. Evidently, this was also the case this study–the principal component analysis shows very close alignment of the polarisability and molecular weight vectors, signifying a high correlation between these parameters (see following section for further discussion, Figure 9). The mean calculated polarisability of all the compounds was found to be 36.3 ± 5.1 Å^3^ with an approximately normal distribution, with a slight right-skew (Table 2, Figure S4). CLCs and flavonolignans have high mean polarisability values (45.6 ± 4.6 Å^3^ and 42.7 ± 1.8 Å^3^, respectively), although dibenzylcclooctadienes had the highest average polarisability (51.2 ± 1.6 Å^3^). Biphenyls and biphenyl ethers had the lowest average polarisabilities. Convention dictates that polarisability values should be ≤68 Å^3^ to be classified as being in KDS [70] all of the compounds in this study met this criterion. 

### 3.2. PCA (Principal Component Analysis)

After looking at the individual molecular descriptors separately, it was decided to conduct an overall analysis using PCA (Principal Component Analysis). PCA is a statistical technique that transforms the data into a series of new, uncorrelated, dimensions called principal components. These principal components are made up by a combination of the variables studied (in this case, the molecular descriptors). Conducting PCA and analysing these principal components allows for the in-depth, simultaneous investigation of all the descriptors, their interactions and interrelationships. Furthermore, PCA can be used to discover groupings of samples–in this study, compounds—and identify the variables that distinguish these clusters of compounds. 

A way to view and analyse the results of a PCA is to plot the principal components against each other, producing a biplot (see Figure 9). In the biplot, the vectors/arrows indicate the direction of influence for each molecular descriptor studied and the data points represent each molecule studied. The biplot given shows the first two principal components–the first principal component is displayed on the x-axis and is the principal component that explains the highest amount of variability of the data, while the second principal component is the dimension in which the data is second-most variable. 

In the PCA for this study, nearly half of the variability in the data set is explained by principal component one while principal component two accounts for ~20% of the variability. Thus, these first two principal components account for ~70% of the variability seen in the data which means that the PCA is an effective technique for this investigation, that captures much of the differences and similarities of the compounds in terms of their molecular descriptors and can provide many useful conclusions and observations. 

**Figure 10:**
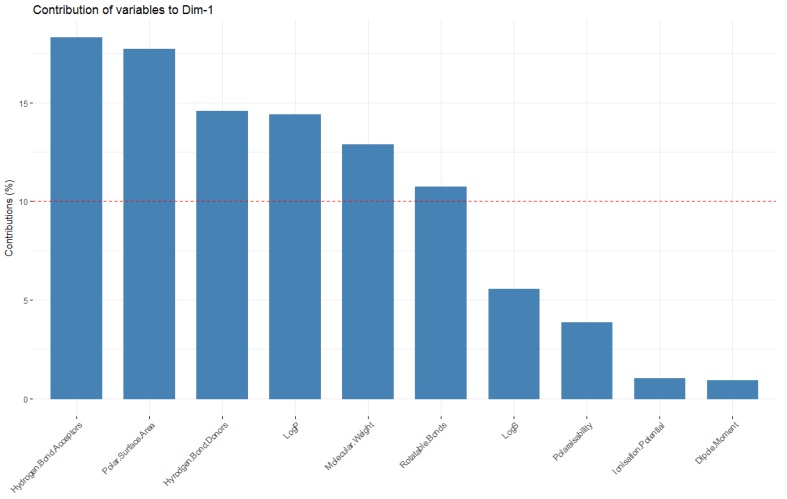
Representation of the contributors to the first principal component.

Looking at the molecular descriptors, it can be seen that dimension one (corresponding to principal component one–the dimension in which data has the greatest variability; x-axis) is largely influenced by the LogP values, number of hydrogen bond donors, acceptors, rotatable bonds and polar surface area—Figure 9 shows these variables having a significant horizontal component to their direction, with Figure 10 quantifying this contribution. The molecular weight of the compound is a significant contributor to dimension one, but also strongly influences principal component two (y axis, as it also has a large vertical component to its direction), along with the polarisability, ionisation potential, dipole moment and LogS. 

It can also be seen from this PCA, that the greater the LogP value (i.e., the greater the lipophilicity), the lower the number of hydrogen bond donors, acceptors and polar surface area for these compounds, as these vectors are in opposite direction to the LogP, signifying an inverse relationship. This coincides with what one would anticipate—compounds with greater hydrogen bond donors, acceptors and polar surface area are expected to be less lipophillic and have lower LogP values. Furthermore, the number of hydrogen bond donors, acceptors and polar surface area are highly correlated, as is signified through them acting in very similar directions in the biplot. This again concurs with what one would expect–a compound with the more hydrogen bond donors and acceptors would be envisaged to have a greater polar surface area. As mentioned when these variables were discussed, ionisation potential, dipole moment and LogS of a compound are shown to be highly correlated. Additionally, LogS is aligned in the opposite orientation to the LogP vector–expected for these two inversely-related measures. 

It should also be noted that the variables that are the highest contributors to the first principal component (number of hydrogen donors, acceptors, rotatable bonds, LogP, molecular weight and polar surface area) are those that are considered when defining chemical spaces (Figure 10, Table 1). 

Where the points are situated on a PCA plot, their relative positions, are a culmination of their values for the various parameters and any observed groupings indicate similarities in the physicochemical properties between compounds in that group. It is apparent that PCA with the ten aforementioned descriptors are able to separate the compounds into groups–classical lignans and neolignans (blue), flavonolignans (green) and CLCs (red). These compounds types are separated on the x-axis–PC1–indicating that these groups can be separated by the variables that contribute to this principal component. Therefore, this analysis can give general indications about the compound classes. In general, it appears that CLCs have higher molecular weights, hydrogen bond acceptors, hydrogen bond donors, rotatable bonds and polar surface areas and lower LogP values (lower lipophilicities). Conversely, classical lignans and neolignans appear to be typified by higher lipophilicities and lower numbers of hydrogen bond acceptors, donors and polar surface areas. In terms of these descriptors, flavonolignans appear to be characterised between these two groups. Additionally, all of the flavonolignans seems to be less variable, while both classical lignans/neolignans and CLCs are significantly more variable. As the CLC group is composed of classical lignan/neolignan types with additional saccharide unit(s), one can see the influence of the inclusion of a sugar moiety, on the molecular descriptors. Furthermore, clemastanin B and secoisolariciresinol diglucoside represented by 155 and 160 in Figure 9, were the two compounds in the study that lie furthest to the right in the biplot shown and were the two compounds in this study that contained two sugar units–this indicates that the number of saccharide units in a CLC can also be differentiated using PCA. 

### 3.3. Lignans in Chemical Space

The proportion of all compounds studied in this investigation that lie within the benchmarks for *lead-like*, *drug-like* and KDS as specified in Table 1 are shown, for each molecular descriptor and when all molecular descriptors are taken into account (Table 3). Immediately apparent is that no compound in this study fits in *lead-like* chemical space, when all of the molecular descriptors that define *lead-likeness* are taken into account. While most compounds have ≤3 hydrogen bond donors and fulfil the requirements for this parameter, the molecular weight, the number of hydrogen bond acceptors and rotatable bonds are the parameters that limit the inclusion of lignans into *lead-like* space. To be *lead-like,* lead structures generally possess low molecular complexity (i.e., low molecular weighs, along with minimal numbers of hydrogen bond acceptors, hydrogen bond donors and rotatable bonds) [56]. Furthermore, these structures are more hydrophyllic and less *drug-like,* hence the strictest criterion for the chemical spaces are those that define *lead-like* space. The purpose of lead structures is to offer a simple scaffold, upon which further complexity can then be added, to provide *drug-like* compounds. Rather than being *lead-like,* the majority of compounds–approximately ¾ of those studied, are *drug-like* in that they already have structures with greater complexity than one would expect from a lead compound. The majority of the studied compounds fulfil the requirements to be considered *drug-like*, thus by definition possess properties and characteristics that indicate they would be appropriate for use as therapeutics. An even higher proportion of the compounds studied are in KDS, thus are in the chemical space that is defined by known drugs.

The success of podophyllotoxin as both a lead compound and as a drug were discussed previously–podophyllotoxin itself is an approved therapeutic for genital warts and associated ailments, while its structurally-related derivatives are clinically approved cancer treatments. Podophyllotoxin is an aryltetralin lactone and as such can be classified as a dibenylbutryolactone or an aryltetralin. For the purposes of this study, it was classified as a dibenzylbutyrolactone as it was apparent that its molecular descriptors and structural scaffold were most similar to compounds of this type. Looking at its molecular parameters, podophyllotoxin meets all the requirements of the Lipinski’s rule of five, with a molecular weight =414.4 gmol^−1^, one hydrogen bond donor, eight hydrogen bond acceptors, LogP = 2.31, PSA = 98.3 Å^2^ and only four rotatable bonds, hence meets all the requirements of being *drug-like*, and therefore it is no surprise that it is an effective medicine. Furthermore, podophyllotoxin has values for the other measures of oral availability, namely LogS, ionisation potential and dipole moment, that signify it to be readily orally-available–a positive and desirable feature for therapeutics. 

Podophyllotoxin is an excellent example of the potential of *drug-like* lignans for use a medicines. Another example of a lignan currently in use is a dibenzylbutane, masoprocol, a form of dihydroguaiaretic acid (Figure 1). Masoprocol is a lipoxygenase inhibitior and is an antineoplastic medicine that is indicated to treat skin growths that result from exposure to the sun [21,75,76]. Like podophyllotoxin, masoprocol fulfils all the criterion that dictate the requirements of a *drug-like* compound. 

Over ¾ of the compounds included in this study, including podophyllotoxin and masoprocol, exist in *drug-like* chemical space, exhibiting properties that allow them to be considered *drug-like* compounds and more likely to be successful therapeutics. There are numerous other lignans that also exhibit potent biological activities and meet all the stipulated benchmarks to be considered *drug-like*, such examples include arctigenin [77], matairesinol [78], sesamin [79] and schizandrin A (Figure 1) [80]. These compounds are just a few of the many hundreds of compounds yet to be fully explored, highlight the vast prospects that lignans provide in medicinal chemistry.

### 3.4. Classical Lignans and Neolignans

**Figure 11:**
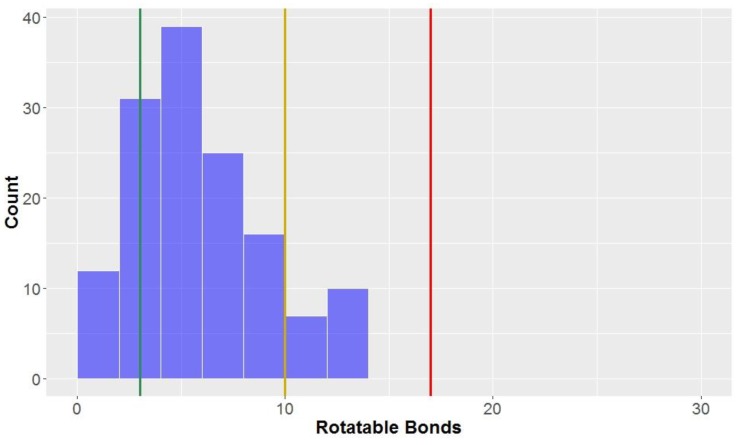
The statistical distribution of the rotatable bonds of the classical lignans and neolignans (green = 3, compounds < 3 are in the lead-like space; yellow = 10, compounds < 10 are in the drug-like space; red = 17, compounds < 17 are in the known drug space). Total number of compounds = 140.

There were 140 classical lignans and neolignans in this study which included the ten most well-known (based on number of references for each compound) of each type. Summary statistics for each of the parameters for the classical lignans/neolignans grouped together, and separate are given in Table 2. Statistical distributions of each of the molecular parameters studied for the classical lignans and neolignans are given (Figure 11, Figures S5–S13) and analysis of their positions in chemical spaces are provided (Table 4, Tables S1–S15). 

While some of the compound sub-classes are very similar in the characteristics studied, there are some compound types that are notably different for various parameters. The compound class that most frequently had markedly higher/lower averages than other lignans/neolignans for the molecular descriptors was the dibenzocyclooctadienes (e.g., (+)-Schizandrin A, Figure 1). Dibenzocyclooctadienes appear to have relatively high molecular weights, polarisability, polar surface area and lipophilicity (LogP), while conversely having relatively low dipole moments and water solubility (LogS). Cyclobutanes and substituted tetrahydrofurans also had high lipophilicities (LogP) and low hydrophillicity measures (LogS), while substituted tetrehydrofurans also had high polar surface areas and lower numbers of rotatable bonds. Arylnapthalenes and 2,6-diarylfurofurans are highly fused scaffolds and this is reflected in their lower rotatable bonds count, compared to other classical lignans and neolignans. Conversely, alkyl aryl ethers and dibenzylbutanes have a less rigid/cyclic structural motif, hence have more rotatable bonds. As well as having a lower amount of rotatable bonds, 2,6-diarylfurofurans also exhibit lower lipophilicities and a lower average dipole moment. Dibenzylbutyrolactones also have lower LogP values and higher water solubilities (LogS), along with higher polar surface areas and dipole moments. Biphenyl ethers are another type that have higher relative calculated dipole moments, and along with biphenyls have lower average molecular weights and polar surface areas. Benzofurans, 1,4-benzodioxanes, 8-1′-bicyclo-[3.2.1]octanes and 8-3′-bicyclo[3.2.1]octanes were compound types that were not notably higher/lower than other classical lignans and neolignans for the parameters investigated.

Overall, as stated previously, none of the classical lignans/neolignans in this study fulfil all the requirements to be considered *lead-like*, although almost all (86.4%) are within the limits that define *drug-like* space and all lignans are in KDS (Table 4). 

The *lead-like* benchmarks that classical lignans and neolignans are least-frequently able to realise are those concerning molecular weight (≤300 g mol^−1^; Figure S5), number of hydrogen bond acceptors and the number of rotatable bonds (both ≤3; Figure S8 and Figure 11). In view of the biosynthesis and definition of lignan structures, it is not surprising that very few of the compounds meet the individual requirements for these parameters, and no compounds are able to meet them all collectively. By definition, lignans and neolignans are the product of an oxidative dimerisation of two or more phenyl propanoid units, which alone would have a weight of at least 240 g mol^−1^. These phenyl propanoid units are almost always oxygenated, often containing several oxygen-containing substituents; the inclusion of more than three oxygen atoms, as is frequently the case with naturally-occurring lignans, would not only increase the molecular weight above the cut-off for *lead-likeness*, but would also exceed the number of allowable hydrogen bond acceptors. 

**Figure 12:**
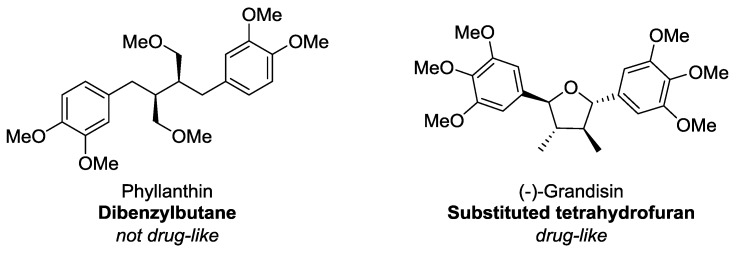
Structures of phyllanthin and (−)-grandisin.

Conversely, it is apparent that lignans and neolignans are generally very *drug-like* and all are within KDS (Table 4). The most discerning *drug-like* space parameter that ~13.5% of the compounds violated were the number of rotatable bonds (criteria: ≤10 rotatable bonds, Figure 11). Related to this, from this study, it can be stated that dibenzylbutanes (e.g., phyllanthin, Figure 12) are the least *drug-like* of all the lignan sub-classes, largely owing to their high number of rotatable bonds–of the ten dibenzylbutanes in this study, six are considered *undrug-like*. In contrast, compounds with similar functional groups but having a more-fused ring scaffold (e.g., (−)-grandisin, Figure 12) are more likely to be drug-like. In all other aspects lignans, in general, almost always fulfil every other requirement that defines *drug-like* space and in several groups, namely dibenzylbutyrolactones, arylnapthalenes/aryltetralins, substituted THFs, 2,6-diarylfurofurans, benzofurans, 1,4-benzodioxanes, 8-1′-bicyclo[3.2.1]octanes, 8-3′-bicyclo[3.2.1]octanes and biphenyl ethers, all members were considered *drug-like*. The high-proportion of *drug-likeness* of classical lignans and neolignans, particularly these aforementioned sub-classes, is a very notable and promising observation that promotes the justifiability and importance of investigating lignans as drugs. 

Considering substituents on these core lignan scaffolds—while for most of the studied descriptors, the most *drug-like* compounds would be those with no/few substituents on the core structures which results in lower values for almost all of the parameters–this, however, is a rarity amongst naturally-occurring lignans and it is very likely that the lipophilicity would increase, possibly beyond acceptable levels. The addition of large polar groups (i.e., sugar moieties as for the CLC’s, see Section 3.6) do have a significant effect on many of the properties of lignans, that largely result in their exclusion from *drug-like* chemical space. In contrast, it can be seen that many of the commonly-occurring, smaller, oxygenated substituents that feature in naturally-occurring lignans (i.e., hydroxy, methoxy and methylenedioxy groups) are well-tolerated within *drug-like* chemical space. This is evidenced by the fact that the parameters (i.e., molecular weight, lipophilicity, hydrogen bond donors/acceptors and polar surface area) that would be most affected by the inclusion of these moieties are very rarely (<1% of all lignans in the study) exceeded. It can therefore be said, that naturally-occurring lignans do have an excellent balance of parameters and structures closely analogous to these, with similar levels of substitution would be of interest. 

Furthermore, as noted when discussing the individual molecular parameters, oral availability can be linked to the dipole moment (ideally < 13 D), LogS (ideally between 0 and −7) and ionisation potential (ideally between 8 and 10 eV). All classical lignans and neolignans had dipole moments under the threshold of 13 D (Figure S10), while all but two and five of the 140 classical lignans and neolignans had an ionisation potential and LogS within the above ranges, respectively (Figures S11 and S12). This is predicates lignans and neolignans to have excellent oral bioavailability–an extremely desirable trait of drugs.

### 3.5. Flavonolignans

As their name suggests, flavonolignans are a structurally very similar to classical lignans and neolignans, however while lignans are formed through the oxidative dimerisation of two or more phenyl propanoid units, the biosynthetic precursors of flavonolignans are a phenyl propanoid unit and a flavone [81]. Flavonolignans are of particular interest to many, owing to their potent biological activities that have been utilised worldwide, for millennia, particularly in the form of silymarin. Silymarin (commonly known as milk thistle extract) is isolated from the seeds of milk thistle, *Silybum marianum*, and is a complex mixture of, predominantly flavonolignan, compounds [82]. Silymarin is a popular liver protectant that has been used in traditional medicine for centuries and is commonly available and used in present-day society [83,84]. 

**Figure 13:**
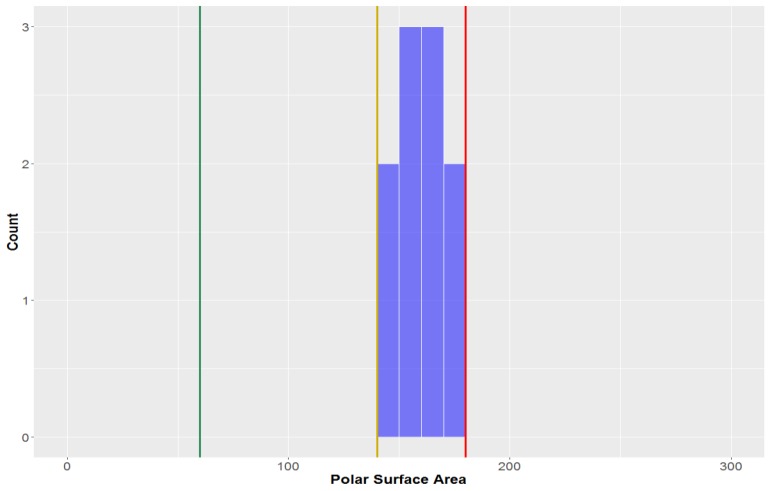
The statistical distribution of the polar surface area (PSA) of the flavonolignans (green = 60, compounds < 60 Å^2^ are in the *lead-like* space; yellow = 140, compounds < 140 Å^2^ are in the *drug-like* space; red = 180, compounds < 180 Å^2^ are in the KDS). Total number of compounds = 10.

Studying the earlier PCA, it is apparent through the close proximity of all flavonolignans in the biplot, that they are all very structurally similar (Figure 9). Furthermore, it can be seen that while flavonolignans are structurally similar to lignans, they are able to be separated on the basis of their molecular descriptors. Flavonolignans are clustered to the right of almost all classical lignans/neolignans and located higher on the y-axis on the PCA than many. One can use the knowledge in which direction the molecular descriptors hold influence to discuss general trends of this compound type. It can be surmised from the PCA, that flavonolignans generally have higher molecular weights, polarisability, number of hydrogen bond donors and acceptors. They also appear to have lower lipophilicities (LogP). These observations are further corroborated by the results in Table 2 (see Figure 13, Figures S14–S22 for analysis of each of the studied molecular descriptors for flavonolignans alone). 

The ten studied flavonolignans were also assessed in relation to the various criterion that define the *lead-like*, *drug-like* and known drug spaces (Table 5). It is notable that flavonolignans are less *drug-like* than classical lignans and neolignans, with no flavonolignans fulfilling all the requirements for *drug-likeness*. The only constraint that flavonolignans exceeded was the polar surface area–no flavonolignans had a PSA ≤ 140 Å^2^ and the mean PSA was 158.5 Å^2^ (Figure 13). It should be noted, however, that all of the compounds had a PSA within the realm of KDS, and the flavonolignans were all in KDS when considering all molecular descriptors.

Reviewing the additional gauges of oral availability; all flavonolignans meet the benchmarks set for the dipole moment, LogS and ionisation potential, thus there is strong indication that flavonolignans are orally available. 

### 3.6. CLCs; Carbohydrate-Lignan Conjugates

**Figure 14:**
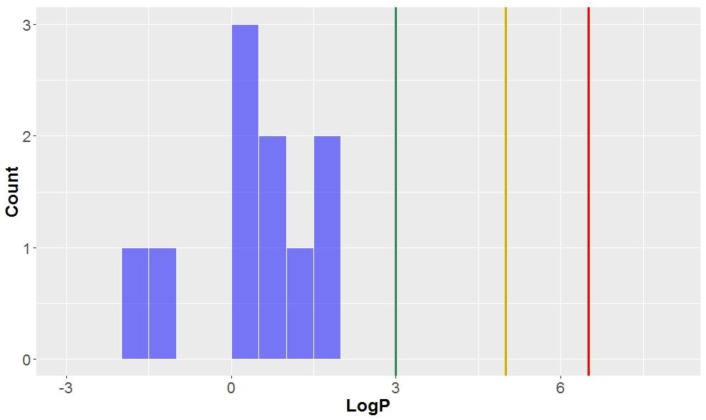
The statistical distribution of the octanol–water partition coefficient (LogP) of the CLCs (green = 3, compounds < 3 are in the *lead-like* space; yellow = 5, compounds < 5 are in the *drug-like* space; red = 6.5, compounds < 6.5 are in the KDS). Total number of compounds = 10.

As the CLCs included in this study are saccharide-containing representatives of various types of classical lignans and neolignans, the differences seen in the CLCs from lignans are due to the sugar moiety. Through the principal component analysis, it was shown that CLCs can be differentiated from both classical lignans/neolignans and flavonolignans on the basis of the molecular descriptors that were included in this study (Figure 9). Analysis of the PCA suggests that the inclusion of a saccharide unit to a lignan increases its mass, number of hydrogen bond donors, acceptors and number of rotatable bonds, as well as its polarisability. They are also indicated to have lower lipophilicities (LogP, Figure 14), and as shown through the comparison of means, a higher affinity for water and other aqueous systems (Table 2, see Figure 14, Figures S23–S31 for the distributions of each of the molecular descriptors).

The low calculated lipophilicities of CLCs are an asset in defining this compound type in chemical space, with all of the CLCs studied having sufficiently low LogP values to be considered *lead-like* (Figure 14), although interestingly this is the only parameter that any CLCs do not exceed for *lead-likeness–*CLCs meet none of the other *lead-like* criteria (Table 6). The molecular descriptor that was the most discriminating for CLCs was they number of hydrogen bond acceptors–no CLCs had sufficiently low enough number of hydrogen bond acceptors to be considered either *lead-like* or *drug-like* (Figure S25)*.* Furthermore, only six out of ten CLCs in this study were in KDS for this parameter. For the other molecular descriptors, very few compounds fell within the discerning bounds of *drug-like* space, less than half of the CLCs within the limits for *drug-like* space for molecular weight (Figure S23), number of hydrogen bond donors (Figure S24), number of rotatable bonds (Figure S27) and polar surface area (Figure S26). 

As a rule, CLCs are distinguished as having many alcohol moieties, which entails that they have low lipophilicities and high molecular weights, number of hydrogen bond acceptors, number of rotatable bonds and polar surface area. One must, however, mention that there is supposition that *drug-likeness* is not a suitable measure of saccharides, which are adsorbed through active transport [85]. Saccharide-based drugs are atypical in KDS [59], and this is reflected in only half of the CLCs studied being entirely in KDS. 

## 4. Summary

In this study, 160 lignans and related compounds were analysed to study their physicochemical properties, their general trends and the variability in these parameters between and within compound types. Furthermore, these molecular descriptors allowed for the defining of these compounds in various chemical spaces, particularly to highlight their *drug-likeness.* It was found, that while no compounds in this study fulfilled all the requirements for six key molecular descriptors to be considered to be *lead-like*, over 3/4 of the compounds were deemed to be in the *drug-like* space and nearly all (~97.5%) were in the KDS. These results strongly advocate for the *drug-likeness* of the majority of lignan compounds that should be further investigated as potential therapeutics. Notably, all compounds from dibenzylbutyrolactones, arylnapthalenes/aryltetralins, substituted THFs, 2,6-diarylfurofurans, benzofurans, 1,4-benzodioxanes, 8-1′-bicyclo[3.2.1]octanes, 8-3′-bicyclo[3.2.1]-octanes and biphenyl ethers sub-classes were shown to *drug-like*, indicating that these sub-classes, in particular, should be further studied for their potential as therapeutic agents.

A PCA analysis of the molecular descriptors particularly highlighted the complex inter-relationships between these physicochemical properties–while the number of hydrogen bond donors, acceptors, rotatable bonds and polar surface area appear to be strong, positive relationship, they collectively have an inverse relationship with the lipophilicity (LogP) of compounds. The first principal component is the dimension that accounts for the greatest variability in the data and it was found that the largest contributors to this principal component were the variables that are considered when defining the chemical spaces. PCA was also able to separate the groups of lignans (classical lignans and neolignans), flavonolignans and CLCs–it was shown that flavnonolignans are more similar to lignans, than their sugar-derivatives, with this separation being distinctive along the first principal component. The differences seen between the different groups in the PCA were also reflected in the differing proportions of lignans, flavonolignans and CLCs that were included in the chemical spaces, particularly in the *drug-like* chemical space. Lignans were almost all (86.4%) *drug-like* and all were in KDS, whereas no flavonolignans or CLCs were *drug-like.* All flavonolignans were in KDS and only half of the CLCs were in KDS. This suggests that lignans, in general, have an excellent balance of the often-paradoxical molecular properties, allowing them to be considered to be *drug-like*, whereas flavonolignans and CLCs have larger values for the variables other than lipophilicity (i.e., polar surface area, number of rotational bonds, hydrogen bond donors and acceptors, as they are further to the right of the PCA plot) that exclude them from *drug-like* chemical space. This is particularly evident for the CLCs, where LogP was the only parameter that all CLCs met the requirements for, to be classified as present in *drug-like* chemical space. 

Within the lignans, there were marked differences between different compound types, with dibenzylcyclooctadienes proving to be the most distinctive compound type, exhibiting relatively high molecular weights, polarisability, polar surface area and lipophilicity (LogP), while conversely having relatively low dipole moments and water solubility (LogS). Overall, the results presented here demonstrate that lignans are very *drug-like*. Coupled with their potent biological activities, their physicochemical properties indicate there is significant value in their study as promising future drug leads. 

## Figures and Tables

**Table 1:** Definition of *lead-like*, *drug-like* and known drug space (KDS) in terms of molecular descriptors.

Descriptor	*Lead-Like* Space	*Drug-Like* Space	Known Drug Space
Molecular weight (g mol^−1^)	300	500	800
Lipophilicity (Log P)	3	5	6.5
Hydrogen bond donors	3	5	7
Hydrogen bond acceptors	3	10	15
Polar surface area (Å^2^)	60	140	180
Rotatable bonds	3	10	17

**Table 2:** Mean, standard deviation (std dev) and median values of the compound types for the ten molecular descriptors analysed in this study.

**Compound Type**	**Molecular Weight (g mol^−1^)**			**Lipophilicity (LogP)**			**Hydrogen Bond Donors**		
	**Mean**	**Std Dev**	**Median**	**Mean**	**Std Dev**	**Median**	**Mean**	**Std Dev**	**Median**
Overall	381.5	70.4	372.4	3.0	1.3	3.1	1.8	1.9	2.0
Classical lignans and neolignans	361.2	42.3	364.4	3.3	1.0	3.4	1.4	1.3	1.0
Flavonolignans	478.6	7.0	482.4	1.6	0.6	1.5	4.1	0.7	4.0
CLCs	567.4	67.5	534.6	0.4	1.1	0.5	6.1	2.1	5.5
Dibenzylbutanes	354.8	36.1	346.4	3.2	1.1	3.2	2.5	1.5	2.0
Dibenzylbutyrolactones	369.6	31.4	372.4	2.5	0.7	2.5	1.6	0.8	2.0
Arylnapthalenes/Aryltetralins	348.2	23.6	350.3	3.1	0.8	3.1	0.7	0.9	0.0
Dibenzocyclooctadienes	413.3	17.8	416.5	3.9	0.7	3.7	0.7	0.8	0.5
Substituted tetrahydrofurans	362.2	28.1	350.4	3.6	0.5	3.6	0.7	0.9	0.0
2,6-Diarylfurofurans	377.8	24.2	371.4	2.7	0.6	2.9	1.0	0.9	1.0
Benzofurans	351.2	21.6	352.4	3.4	1.1	3.1	2.1	1.3	2.5
1,4-Benzodioxanes	352.0	37.3	359.4	3.3	1.2	3.5	1.6	1.4	1.5
Alkyl aryl ethers	387.0	15.5	377.4	3.1	1.4	2.7	2.4	1.8	3.0
Biphenyls	313.8	62.6	298.4	3.5	1.6	3.9	2.4	1.5	2.0
Cyclobutanes	371.6	51.1	372.4	3.7	0.9	3.9	1.0	1.4	0.0
8-1′-Bicyclo[3.2.1]octanes	377.8	15.3	373.4	3.3	0.6	3.2	0.4	0.8	0.0
8-3′-Bicyclo[3.2.1]octanes	381.4	30.0	386.4	3.5	0.5	3.6	0.8	0.4	1.0
Biphenyl ethers	296.5	27.4	287.3	3.1	1.1	2.6	1.3	0.8	1.5
	**Hydrogen Bond Acceptors**			**Polar Surface Area (Å^2^)**			**Rotatable Bonds**		
	**Mean**	**Std Dev**	**Median**	**Mean**	**Std Dev**	**Median**	**Mean**	**Std Dev**	**Median**
Overall	6.3	3.4	6.0	79.5	37.1	70.2	7.0	3.8	6.0
Classical lignans and neolignans	5.4	1.7	5.8	68.3	22.5	66.9	6.4	3.1	6.0
Flavonolignans	9.0	0.8	9.7	158.5	11.4	159.2	7.1	0.7	7.0
CLCs	16.6	3.8	14.9	156.9	29.3	142.1	15.1	5.0	14.5
Dibenzylbutanes	4.9	1.5	5.0	71.2	22.2	69.1	11.0	1.9	11.0
Dibenzylbutyrolactones	6.5	1.2	6.0	90.8	13.6	87.9	7.6	1.5	8.0
Arylnapthalenes/Aryltetralins	4.7	1.5	5.3	61.6	15.4	69.4	3.4	1.9	3.0
Dibenzocyclooctadienes	5.1	0.8	4.9	51.2	11.9	49.4	5.0	1.6	5.0
Substituted tetrahydrofurans	4.9	0.5	4.7	50.6	12.6	47.8	3.4	1.6	4.0
2,6-Diarylfurofurans	6.8	0.5	6.4	66.3	14.0	66.6	3.3	2.2	4.0
Benzofurans	5.1	1.8	6.4	69.2	25.2	80.7	7.1	2.5	8.5
1,4-Benzodioxanes	5.0	1.4	4.5	70.7	23.8	59.3	5.8	1.7	6.0
Alkyl aryl ethers	6.7	1.6	7.2	76.9	27.8	80.9	12.1	1.3	12.5
Biphenyls	4.1	3.1	2.6	63.2	28.4	56.2	8.4	2.3	7.0
Cyclobutanes	4.5	1.5	4.3	66.8	36.4	59.6	4.6	1.6	5.0
8-1′-Bicyclo[3.2.1]octanes	6.3	0.7	6.0	69.5	8.5	66.5	4.7	1.3	5.0
8-3′-Bicyclo[3.2.1]octanes	6.7	0.8	6.5	71.8	7.6	72.9	5.8	1.4	6.0
Biphenyl ethers	4.1	1.5	4.5	76.1	26.2	84.4	7.1	1.8	7.5
	**Dipole Moment (D)**			**Water Solubility (LogS)**			**Ionisation Potential (eV)**		
	**Mean**	**Std Dev**	**Median**	**Mean**	**Std Dev**	**Median**	**Mean**	**Std Dev**	**Median**
Overall	4.1	1.9	3.9	−4.1	1.4	−4.1	9.0	0.4	9.0
Classical lignans and neolignans	4.1	1.9	4.0	−4.2	1.4	−4.2	9.0	0.4	9.0
Flavonolignans	4.0	2.5	3.2	−4.4	0.8	−4.4	9.1	0.2	9.1
CLCs	4.7	1.9	3.9	−2.5	1.3	−2.4	9.0	0.2	9.0
Dibenzylbutanes	4.1	1.2	3.9	−3.4	1.2	−3.0	9.1	0.2	9.0
Dibenzylbutyrolactones	5.5	2.2	4.7	−2.6	0.6	−2.6	9.2	0.2	9.2
Arylnapthalenes/Aryltetralins	5.1	2.8	4.9	−3.5	1.3	−3.5	8.4	0.2	8.4
Dibenzocyclooctadienes	2.6	1.0	2.5	−5.6	0.9	−5.4	8.6	0.3	8.5
Substituted tetrahydrofurans	3.5	1.6	3.8	−5.4	1.1	−5.0	9.0	0.3	9.0
2,6-Diarylfurofurans	2.7	1.3	2.8	−3.9	1.0	−4.4	8.9	0.3	9.0
Benzofurans	3.0	0.9	3.1	−4.7	1.1	−4.4	8.7	0.2	8.7
1,4-Benzodioxanes	3.9	2.0	3.8	−4.9	1.2	−4.7	9.0	0.2	9.1
Alkyl aryl ethers	4.0	1.5	4.4	−4.2	1.9	−3.2	9.1	0.1	9.1
Biphenyls	3.6	1.7	3.5	−3.5	0.9	−4.0	8.7	0.2	8.8
Cyclobutanes	3.7	1.8	3.9	−5.8	1.5	−5.4	9.2	0.5	9.0
8-1′-Bicyclo[3.2.1]octanes	4.9	1.2	5.2	−3.5	0.5	−3.5	8.9	0.5	8.8
8-3′-Bicyclo[3.2.1]octanes	4.5	1.8	4.5	−4.1	0.7	−4.3	9.0	0.6	8.9
Biphenyl ethers	5.3	2.1	4.9	−3.6	0.6	−3.7	9.6	0.3	9.6
	**Polarisability (Å^3^)**								
	**Mean**	**Std Dev**	**Median**						
Overall	36.3	5.1	36.0						
Classical lignans and neolignans	35.1	4.2	35.1						
Flavonolignans	42.7	1.8	43.2						
CLCs	45.6	4.6	46.5						
Dibenzylbutanes	32.2	4.1	30.4						
Dibenzylbutyrolactones	32.0	2.8	30.9						
Arylnapthalenes/Aryltetralins	32.2	1.7	31.9						
Dibenzocyclooctadienes	51.2	1.6	49.4						
Substituted tetrahydrofurans	38.7	3.5	37.1						
2,6-Diarylfurofurans	37.3	2.8	37.1						
Benzofurans	35.3	1.8	35.1						
1,4-Benzodioxanes	35.7	3.8	35.7						
Alkyl aryl ethers	35.7	3.5	34.0						
Biphenyls	31.0	3.7	29.9						
Cyclobutanes	38.7	4.6	38.1						
8-1′-Bicyclo[3.2.1]octanes	36.3	2.8	36.1						
8-3′-Bicyclo[3.2.1]octanes	37.5	3.0	37.0						
Biphenyl ethers	29.9	1.9	29.6						

**Table 3:** All compounds in this study and their inclusion within the defined chemical spaces.

Overall	*Lead-Like* Space	*Drug-Like* Space	Known Drug Space
Molecular weight (g mol^−1^)	10.6%	94.4%	100%
Lipophilicity (Log P)	46.3%	99.4%	100%
Hydrogen bond donors	81.3%	96.3%	98.8%
Hydrogen bond acceptors	15.0%	93.8%	97.5%
Polar surface area (Å^2^)	33.1%	88.1%	98.8%
Rotatable bonds	12.5%	85.0%	98.1%
All criteria	0.0%	75.6%	97.5%

**Table 4:** All classical lignans and neolignans (not CLCs or flavonolignans) studied within the defined chemical spaces.

Overall	*Lead-Like* Space	*Drug-Like* Space	Known Drug Space
Molecular weight (g mol^−1^)	12.1%	100%	100%
Lipophilicity (Log P)	38.6%	99.3%	100%
Hydrogen bond donors	91.4%	99.3%	100%
Hydrogen bond acceptors	17.1%	100%	100%
Polar surface area (Å^2^)	37.9%	99.3%	100%
Rotatable bonds	14.3%	87.9%	100%
All criteria	0.0%	86.4%	100%

**Table 5:** Flavonolignans studied within the defined chemical spaces.

Overall	*Lead-Like* Space	*Drug-Like* Space	Known Drug Space
Molecular weight (g mol^−1^)	0%	100%	100%
Lipophilicity (Log P)	100%	100%	100%
Hydrogen bond donors	20%	100%	100%
Hydrogen bond acceptors	0%	100%	100%
Polar surface area (Å^2^)	0%	0%	100%
Rotatable bonds	0%	100%	100%
All criteria	0%	0%	100%

**Table 6:** CLCs studied within the defined chemical spaces.

Overall	*Lead-Like* Space	*Drug-Like* Space	Known Drug Space
Molecular weight (g mol^−1^)	0%	10%	100%
Lipophilicity (Log P)	100%	100%	100%
Hydrogen bond donors	0%	50%	80%
Hydrogen bond acceptors	0%	0%	60%
Polar surface area (Å^2^)	0%	20%	80%
Rotatable bonds	0%	30%	70%
All criteria	0%	0%	50%

## Supplementary Materials

The supplementary materials are available online.
