# Supplementary material for: Lignans: A Chemometric Analysis
Source: Molecules. 2018 Jul 9;23(7):1666. doi: 10.3390/molecules23071666 (PMC6099669; doi:10.3390/molecules23071666)
Supplement: Supplementary file 1 [file molecules-23-01666-s001.pdf]

Supporting Information for:

Lignans: A Chemometric Analysis

*Lisa I. Pilkington**

School of Chemical Sciences, The University of Auckland, Auckland, New Zealand.

*To whom correspondence should be addressed: School of Chemical Sciences, The University of Auckland, Private Bag 92019, Auckland 1142, New Zealand.
E-mail: lisa.pilkington@auckland.ac.nz, Tel. 64-9-373-7599 ext. 86776

Table of Contents

Figure S1. The statistical distribution of the dipole moments of all analysed compounds....S4

Figure S2. The statistical distribution of the water solubility (LogS) of all analysed compounds.. ....S4

Figure S3. The statistical distribution of the ionisation potentials of all analysed compounds. S5

Figure S5. The statistical distribution of the molecular weight of all classical lignans and neolignans. S6

Figure S6. The statistical distribution of the octanol – water partition coefficient (LogP) of all classical lignans and neolignans. S6

Figure S7. The statistical distribution of the hydrogen bond donors of classical lignans and neolignans. S7

Figure S8. The statistical distribution of the hydrogen bond acceptors of classical lignans and neolignans. S7

Figure S9. The statistical distribution of the polar surface area (PSA) of classical lignans and neolignans. S8

Figure S10. The statistical distribution of the dipole moments of classical lignans and neolignans. S8

Figure S11. The statistical distribution of the water solubility (LogS) of classical lignans and neolignans. S9

Figure S12. The statistical distribution of the ionisation potentials of classical lignans and neolignans. S9

Figure S13. The statistical distribution of the polarisability of classical lignans and neolignans. S10

Table S1. Dibenzylbutanes studied within the defined chemical spaces. S11

Table S2. Dibenzylbutyrolactones studied within the defined chemical spaces. S11

Table S3. Arylnapthalenes/aryltetralins studied within the defined chemical spaces....S11

Table S4. Dibenzocyclooctadienes studied within the defined chemical spaces. S12

Table S5. Substituted tetrahydrofurans studied within the defined chemical spaces....S12

Table S6. 2,6-Diarylfurans studied within the defined chemical spaces. S12

Table S7. Benzofurans studied within the defined chemical spaces. S13

Table S8. 1,4-Benzodioxanes studied within the defined chemical spaces. S13

Table S9. Alkyl aryl ethers studied within the defined chemical spaces. S13

Table S10. Biphenyls studied within the defined chemical spaces. S14

Table S11. Cyclobutanes studied within the defined chemical spaces. S14

Table S12. 8-1′-Bicyclo[3.2.1]octanes studied within the defined chemical spaces. S14

Table S14. 8-3′-Bicyclo[3.2.1]octanes studied within the defined chemical spaces. S15

Table S15. Biphenyl ethers studied within the defined chemical spaces. S15

Figure S14. The statistical distribution of the molecular weight of the flavonolignans. S16

Figure S16. The statistical distribution of the hydrogen bond donors of flavonolignans....S17
Figure S18. The statistical distribution of the rotatable bonds of the flavonolignans. S18
Figure S19. The statistical distribution of the dipole moment of the flavonolignans..........S18
Figure S20. The statistical distribution of the water solubility (LogS) of the flavonolignans S19
Figure S21. The statistical distribution of the ionisation potentials of the flavonolignans..S19
Figure S22. The statistical distribution of the polarisability of the flavonolignans.............S20
Figure S23. The statistical distribution of the molecular weight of the CLCs......................S21
Figure S24. The statistical distribution of the hydrogen bond donors of the CLCs.. ....S21
Figure S25. The statistical distribution of the hydrogen bond acceptors of the CLCs S22
Figure S27. The statistical distribution of the rotatable bonds of the CLCs........................S23
Figure S28. The statistical distribution of the dipole moments of the CLCs.......................S23
Figure S29. The statistical distribution of the water solubilities (LogS) of the CLCs.........S24
Figure S30. The statistical distribution of the ionisation potentials of the CLCs.. ....S24
Figure S31. The statistical distribution of the polarisabilities of the CLCs.........................S25
Table S16: The details, molecular descriptors and classification for the compounds used in this study. S26

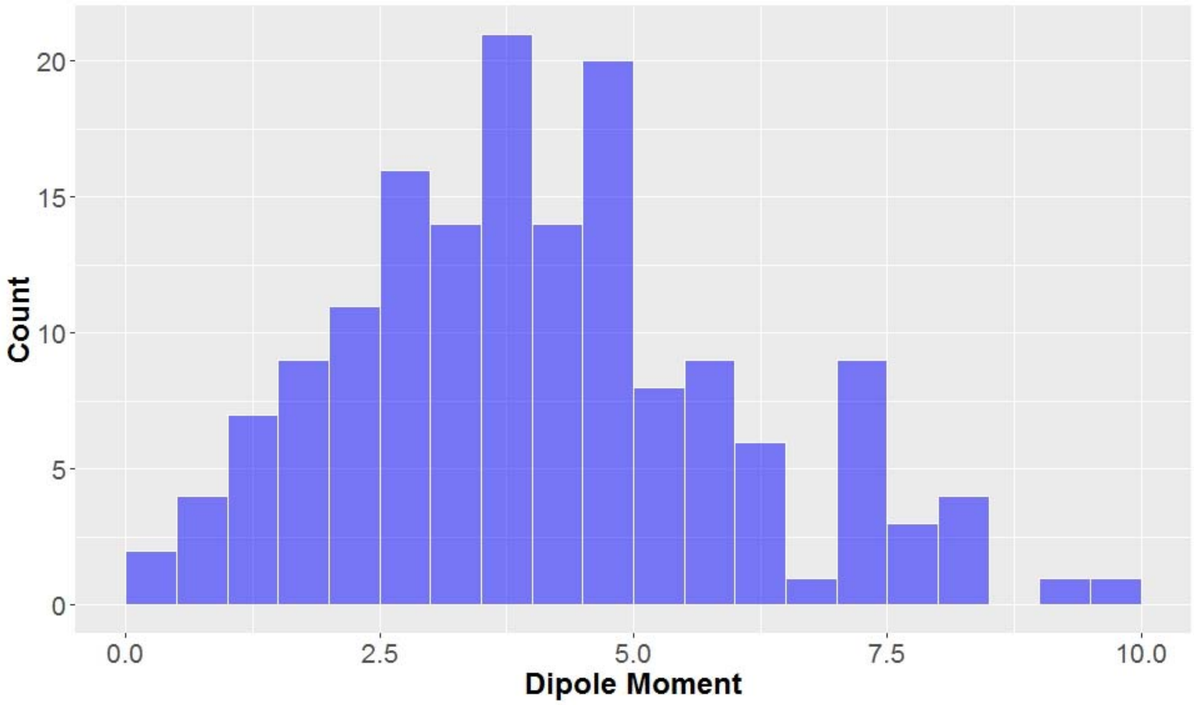

Figure S1. The statistical distribution of the dipole moments of all analysed compounds. Total number of compounds = 160.

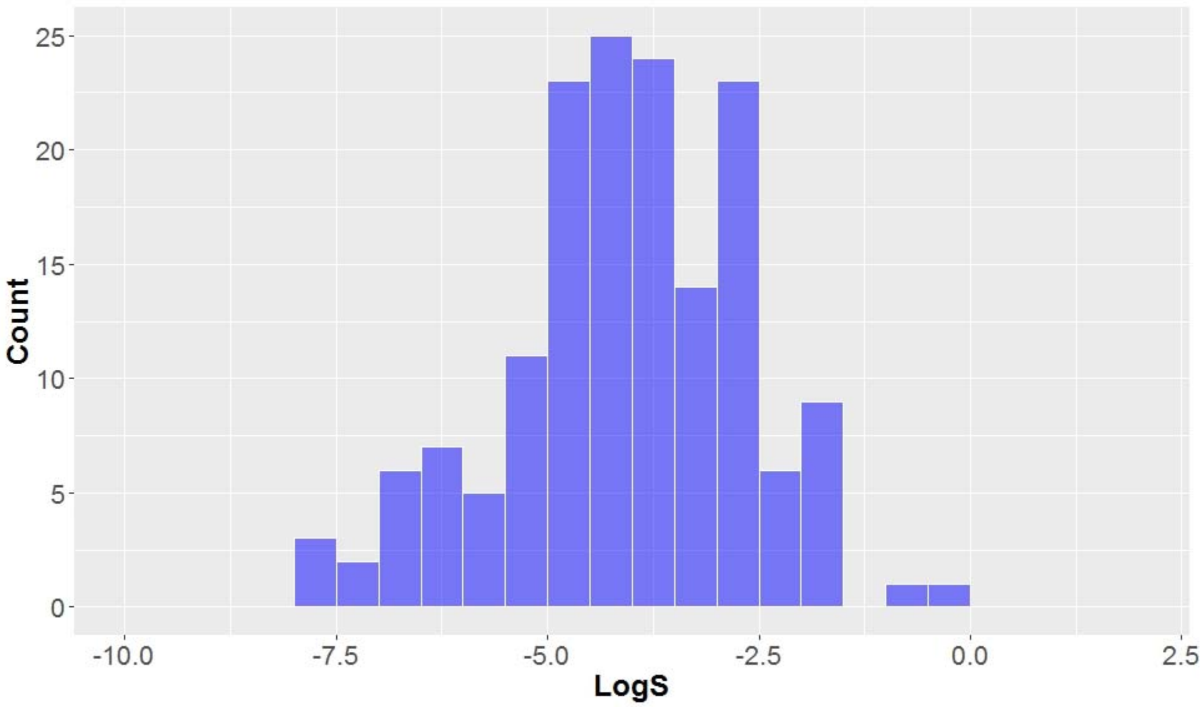

Figure S2. The statistical distribution of the water solubility (LogS) of all analysed compounds. Total number of compounds = 160.

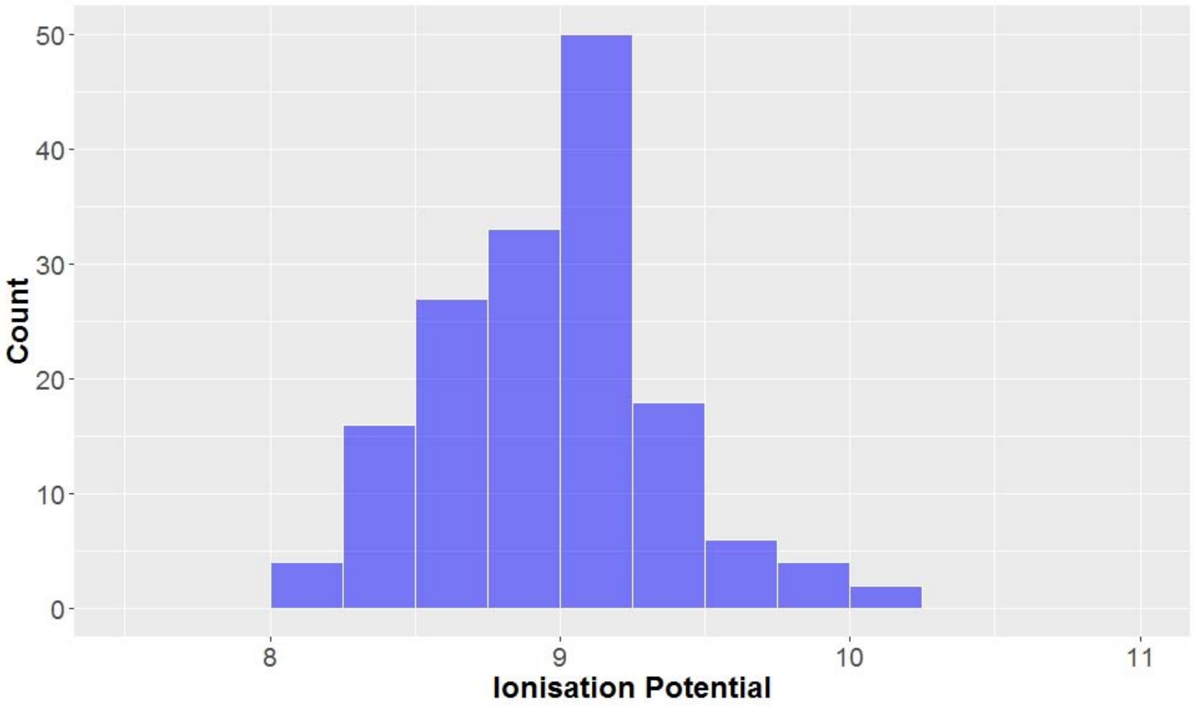

Figure S3. The statistical distribution of the ionisation potentials of all analysed compounds. Total number of compounds = 160.

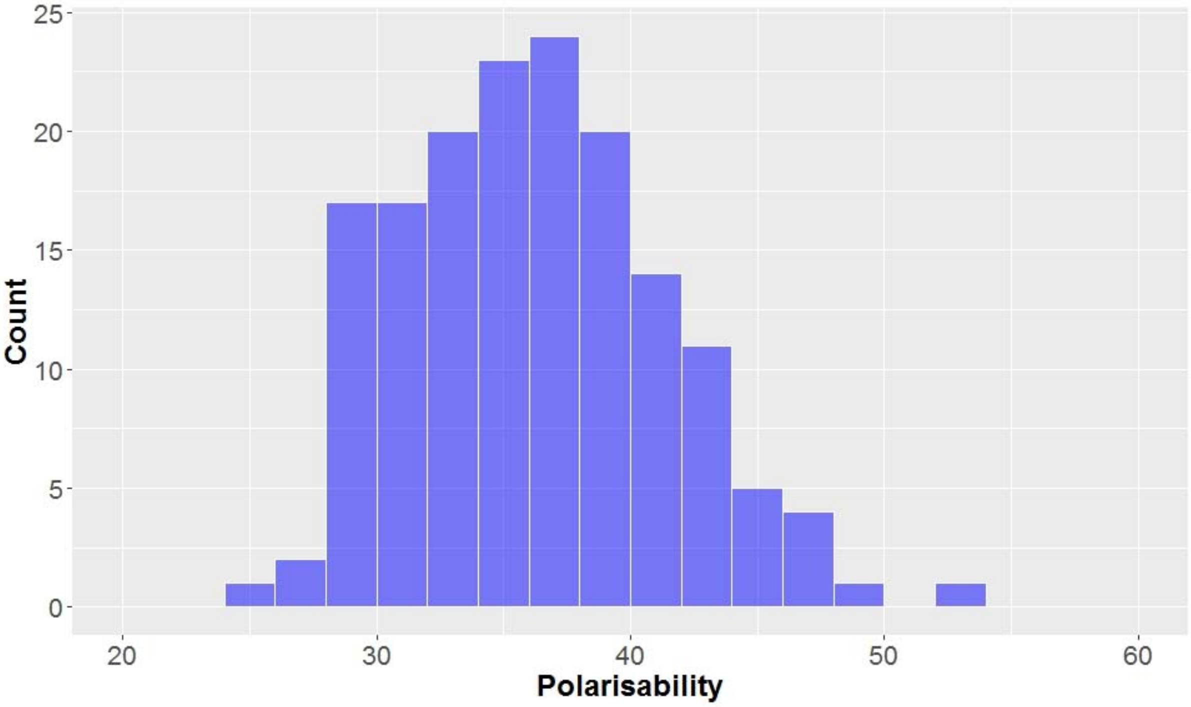

Figure S4. The statistical distribution of the polarisability of all analysed compounds. Total number of compounds = 160.

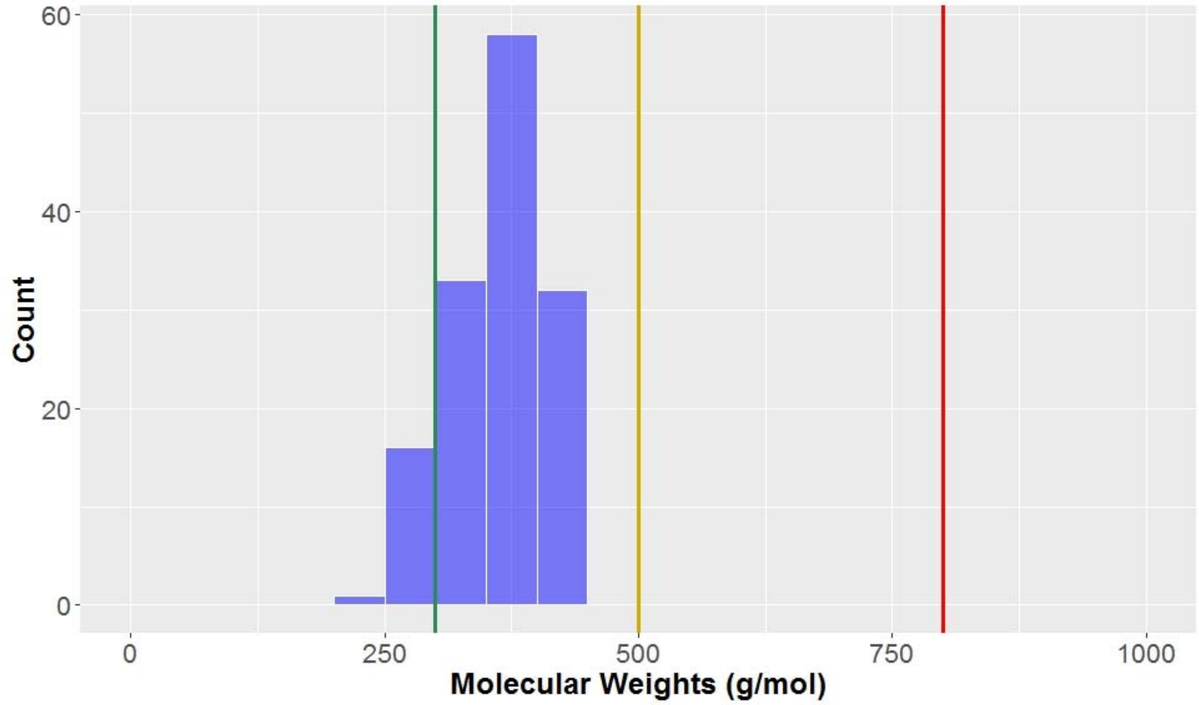

Figure S5. The statistical distribution of the molecular weight of all classical lignans and neolignans (green = 300 g mol^{-1}, compounds < 300 g $mol^{-1}$are in the *lead-like* space; yellow = 500 g mol^{-1}, compounds < 500 g mol^{-1} are in the *drug-like* space; red= 800 g mol^{-1}, compounds < 800 g $mol^{-1}$are in the KDS). Total number of compounds = 140.

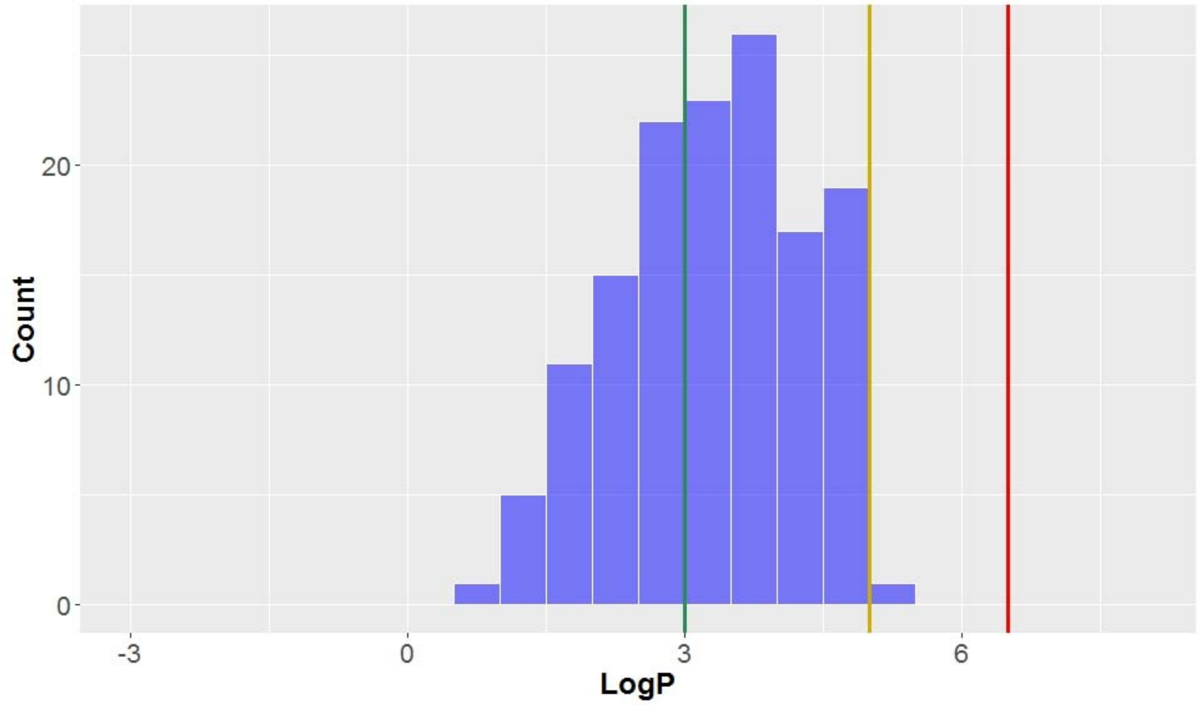

Figure S6. The statistical distribution of the octanol – water partition coefficient (LogP) of all classical lignans and neolignans (green = 3, compounds < 3 are in the *lead-like* space; yellow = 5, compounds < 5 are in the *drug-like* space; red= 6.5, compounds < 6.5 are in the KDS). Total number of compounds = 140.

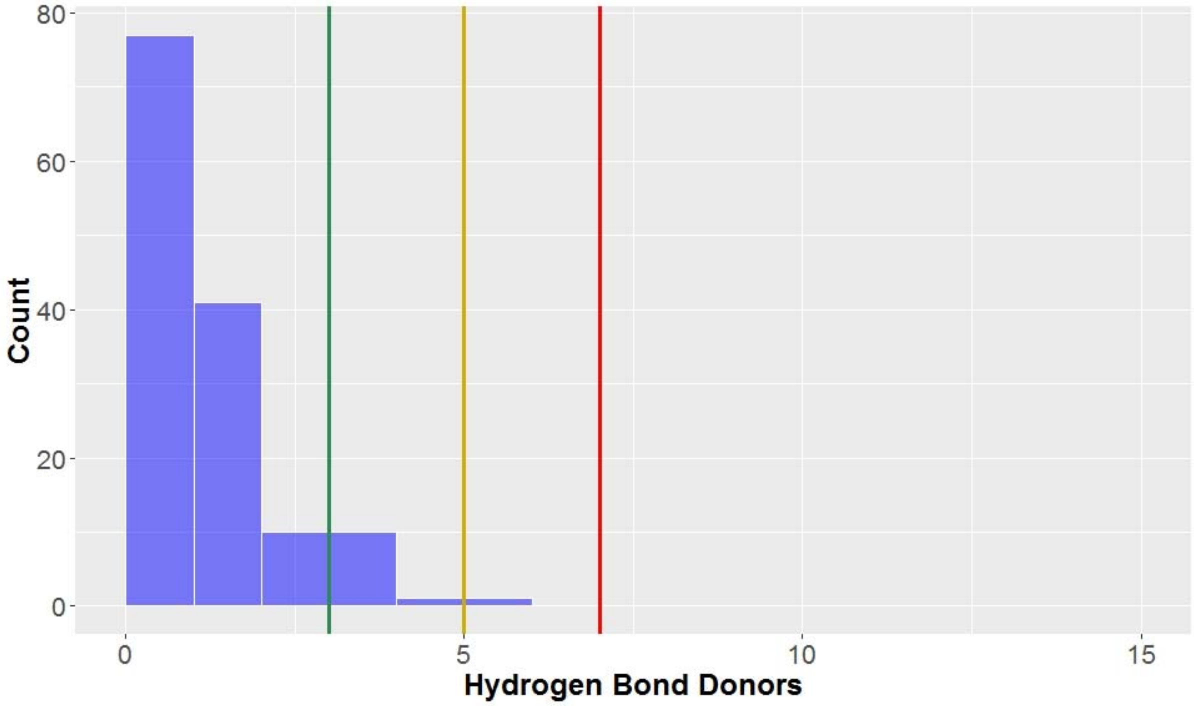

Figure S7. The statistical distribution of the hydrogen bond donors of classical lignans and neolignans (green = 3, compounds < 3 are in the *lead-like* space; yellow = 5, compounds < 5 are in the *drug-like* space; red= 7, compounds < 7 are in the KDS). Total number of compounds = 140.

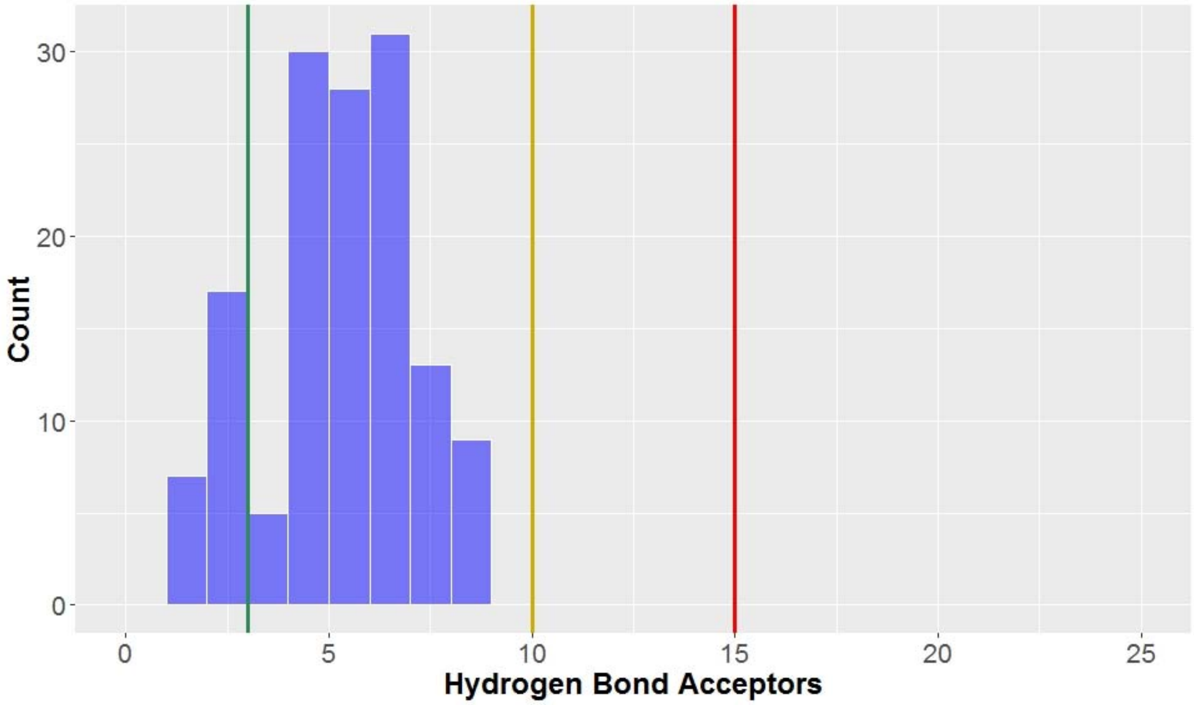

Figure S8. The statistical distribution of the hydrogen bond acceptors of classical lignans and neolignans (green = 3, compounds < 3 are in the *lead-like* space; yellow = 5, compounds < 5 are in the *drug-like* space; red = 15, compounds < 15 are in the KDS). Total number of compounds = 140.

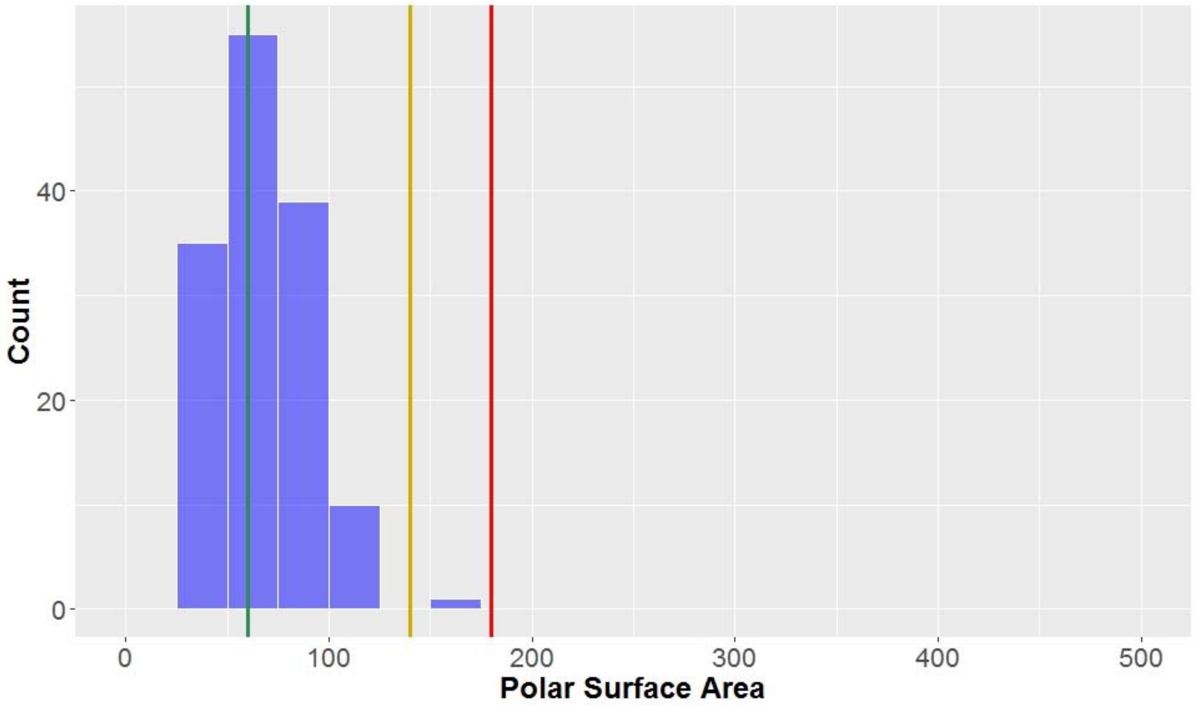

Figure S9. The statistical distribution of the polar surface area (PSA) of classical lignans and neolignans (green = 60, compounds < 60 Å^2 are in the *lead-like* space; yellow = 140, compounds < 140 Å^2 are in the *drug-like* space; red= 180, compounds < 180 Å^2 are in the KDS). Total number of compounds = 140.

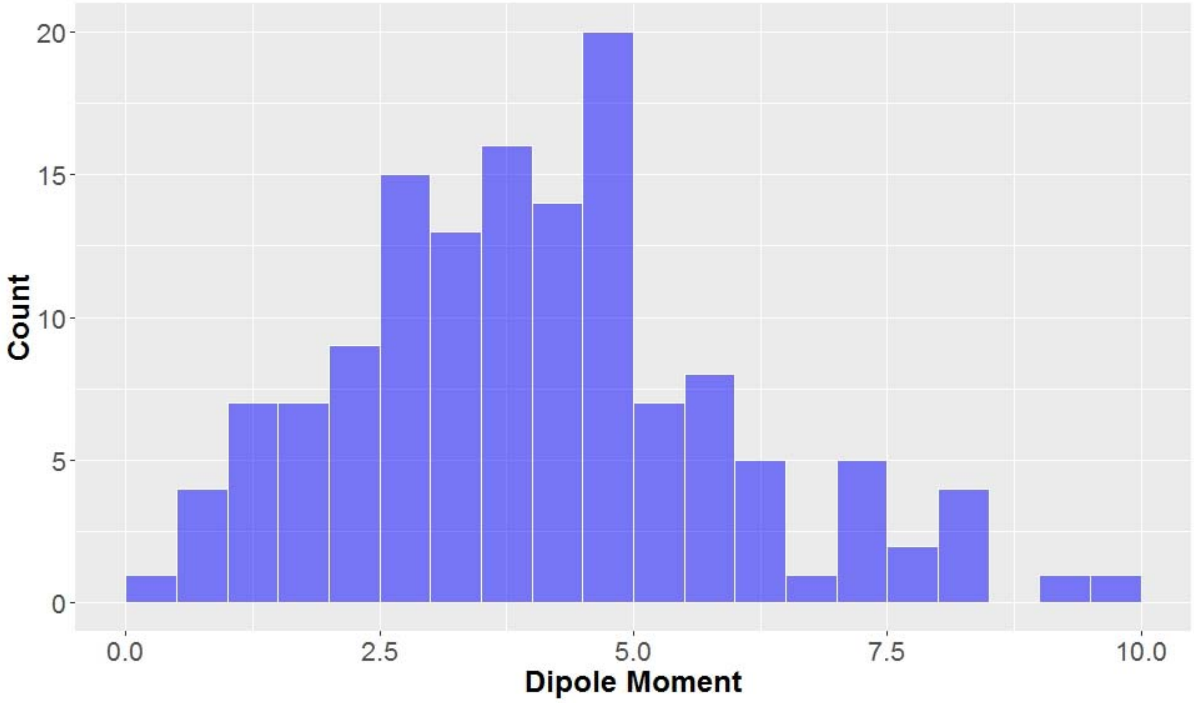

Figure S10. The statistical distribution of the dipole moments of classical lignans and neolignans. Total number of compounds = 140.

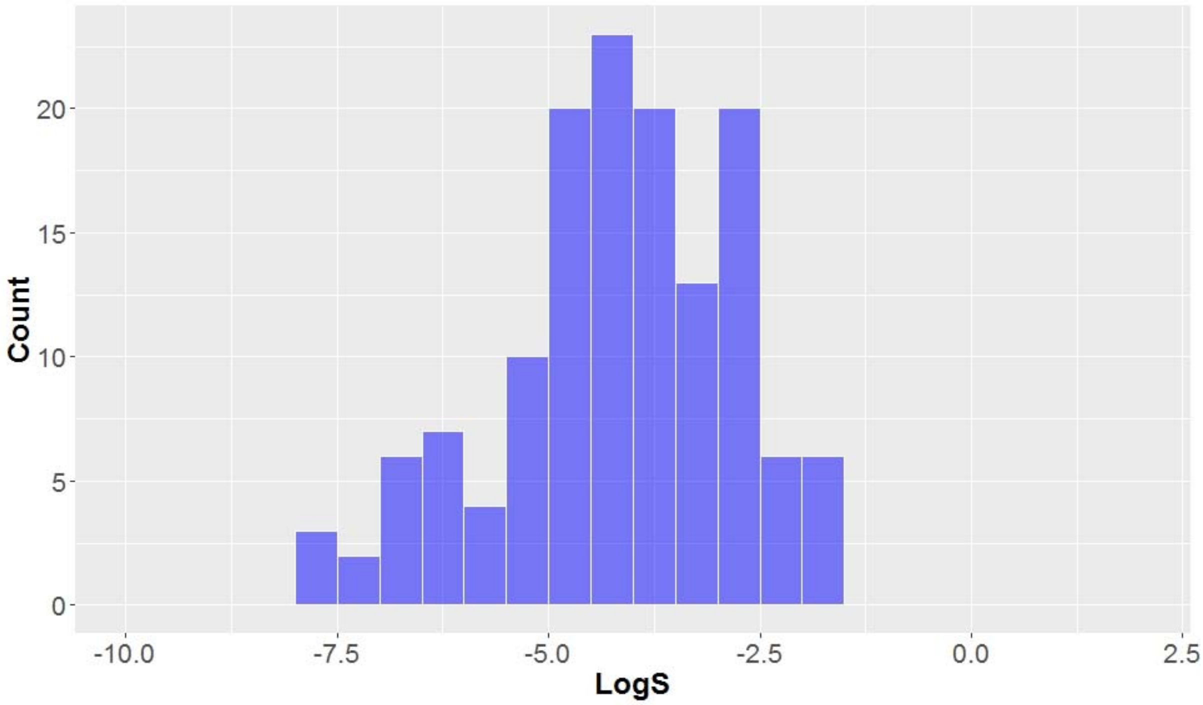

Figure S11. The statistical distribution of the water solubility (LogS) of classical lignans and neolignans. Total number of compounds = 140.

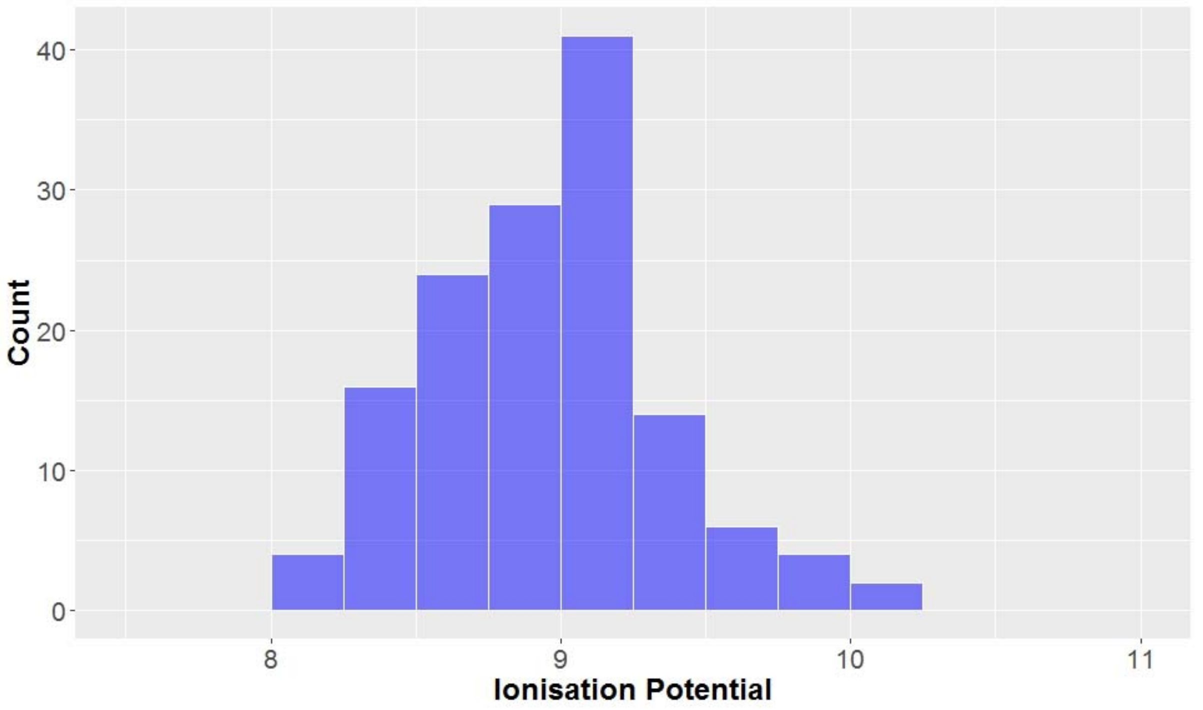

Figure S12. The statistical distribution of the ionisation potentials of classical lignans and neolignans. Total number of compounds = 140.

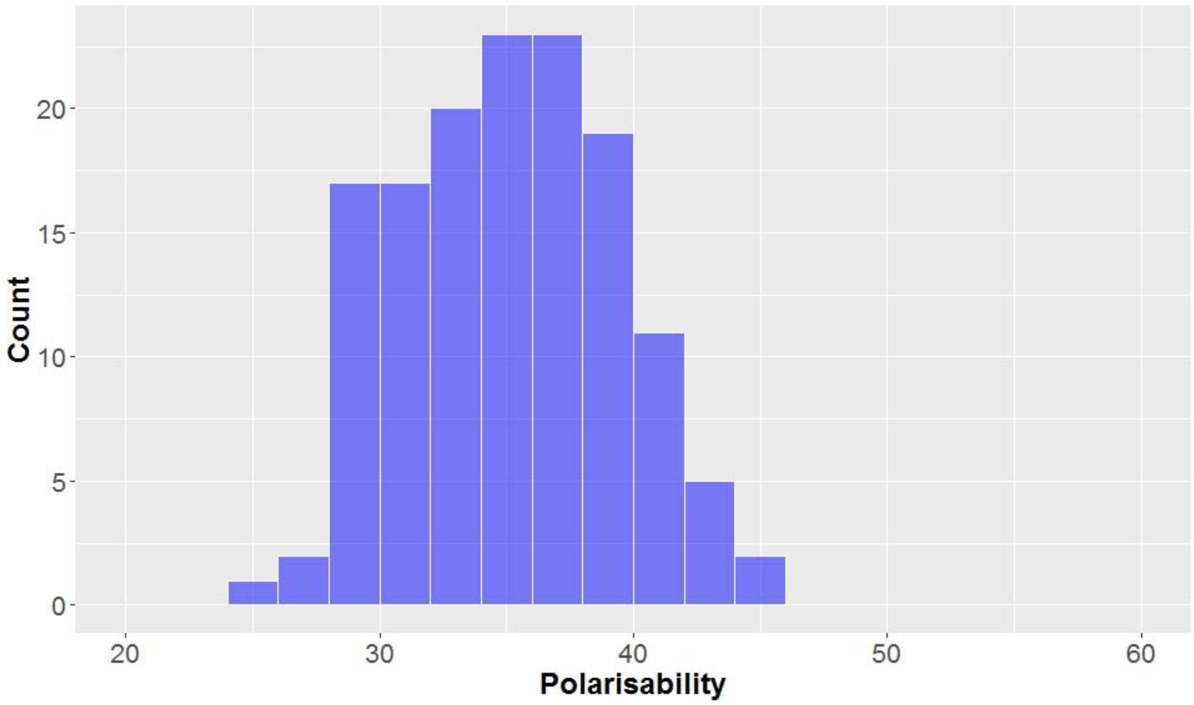

Figure S13. The statistical distribution of the polarisability of classical lignans and neolignans. Total number of compounds = 140.

Table S1. Dibenzylbutanes studied within the defined chemical spaces.

Overall	*Lead-like* Space	*Drug-like* Space	Known Drug Space
Molecular weight (g mol^{-1})	0%	100%	100%
Lipophilicity (Log P)	40%	100%	100%
Hydrogen bond donors	70%	90%	100%
Hydrogen bond acceptors	30%	60%	100%
Polar surface area ($Å^2$)	30%	100%	100%
Rotatable bonds	0%	40%	100%
All criteria	0%	40%	100%

Table S2. Dibenzylbutyrolactones studied within the defined chemical spaces.

Overall	*Lead-like* Space	*Drug-like* Space	Known Drug Space
Molecular weight (g mol^{-1})	10%	100%	100%
Lipophilicity (Log P)	80%	100%	100%
Hydrogen bond donors	100%	100%	100%
Hydrogen bond acceptors	0%	100%	100%
Polar surface area ($Å^2$)	0%	100%	100%
Rotatable bonds	0%	100%	100%
All criteria	0%	100%	100%

Table S3. Arylnapthalenes/aryltetralins studied within the defined chemical spaces.

Overall	*Lead-like* Space	*Drug-like* Space	Known Drug Space
Molecular weight (g mol^{-1})	0%	100%	100%
Lipophilicity (Log P)	50%	100%	100%
Hydrogen bond donors	100%	100%	100%
Hydrogen bond acceptors	30%	100%	100%
Polar surface area ($Å^2$)	40%	100%	100%
Rotatable bonds	60%	100%	100%
All criteria	0%	100%	100%

Table S4. Dibenzocyclooctadienes studied within the defined chemical spaces.

Overall	*Lead-like* Space	*Drug-like* Space	Known Drug Space
Molecular weight (g mol^{-1})	0%	100%	100%
Lipophilicity (Log P)	10%	90%	100%
Hydrogen bond donors	100%	100%	100%
Hydrogen bond acceptors	0%	50%	100%
Polar surface area ($Å^2$)	80%	100%	100%
Rotatable bonds	10%	100%	100%
All criteria	0%	50%	100%

Table S5. Substituted tetrahydrofurans studied within the defined chemical spaces.

Overall	*Lead-like* Space	*Drug-like* Space	Known Drug Space
Molecular weight (g mol^{-1})	0%	100%	100%
Lipophilicity (Log P)	10%	100%	100%
Hydrogen bond donors	100%	100%	100%
Hydrogen bond acceptors	0%	100%	100%
Polar surface area ($Å^2$)	70%	100%	100%
Rotatable bonds	30%	100%	100%
All criteria	0%	100%	100%

Table S6. 2,6-Diarylfurofurans studied within the defined chemical spaces.

Overall	*Lead-like* Space	*Drug-like* Space	Known Drug Space
Molecular weight (g mol^{-1})	0%	100%	100%
Lipophilicity (Log P)	80%	100%	100%
Hydrogen bond donors	100%	100%	100%
Hydrogen bond acceptors	0%	100%	100%
Polar surface area ($Å^2$)	40%	100%	100%
Rotatable bonds	30%	100%	100%
All criteria	0%	100%	100%

Table S7. Benzofurans studied within the defined chemical spaces.

Overall	*Lead-like* Space	*Drug-like* Space	Known Drug Space
Molecular weight (g mol^{-1})	0%	100%	100%
Lipophilicity (Log P)	50%	100%	100%
Hydrogen bond donors	100%	100%	100%
Hydrogen bond acceptors	40%	100%	100%
Polar surface area ($Å^2$)	40%	100%	100%
Rotatable bonds	0%	100%	100%
All criteria	0%	100%	100%

Table S8. 1,4-Benzodioxanes studied within the defined chemical spaces.

Overall	*Lead-like* Space	*Drug-like* Space	Known Drug Space
Molecular weight (g mol^{-1})	20%	100%	100%
Lipophilicity (Log P)	30%	100%	100%
Hydrogen bond donors	90%	100%	100%
Hydrogen bond acceptors	20%	100%	100%
Polar surface area ($Å^2$)	60%	100%	100%
Rotatable bonds	20%	100%	100%
All criteria	0%	100%	100%

Table S9. Alkyl aryl ethers studied within the defined chemical spaces.

Overall	*Lead-like* Space	*Drug-like* Space	Known Drug Space
Molecular weight (g mol^{-1})	0%	100%	100%
Lipophilicity (Log P)	50%	100%	100%
Hydrogen bond donors	50%	100%	100%
Hydrogen bond acceptors	0%	100%	100%
Polar surface area ($Å^2$)	30%	100%	100%
Rotatable bonds	0%	10%	100%
All criteria	0%	10%	100%

Table S10. Biphenyls studied within the defined chemical spaces.

Overall	*Lead-like* Space	*Drug-like* Space	Known Drug Space
Molecular weight (g mol^{-1})	50%	100%	100%
Lipophilicity (Log P)	40%	100%	100%
Hydrogen bond donors	90%	90%	100%
Hydrogen bond acceptors	60%	100%	100%
Polar surface area ($Å^2$)	50%	100%	100%
Rotatable bonds	0%	80%	100%
All criteria	0%	80%	100%

Table S11. Cyclobutanes studied within the defined chemical spaces.

Overall	*Lead-like* Space	*Drug-like* Space	Known Drug Space
Molecular weight (g mol^{-1})	10%	100%	100%
Lipophilicity (Log P)	10%	100%	100%
Hydrogen bond donors	90%	100%	100%
Hydrogen bond acceptors	30%	100%	100%
Polar surface area ($Å^2$)	60%	90%	100%
Rotatable bonds	20%	100%	100%
All criteria	0%	90%	100%

Table S12. 8-1′-Bicyclo[3.2.1]octanes studied within the defined chemical spaces.

Overall	*Lead-like* Space	*Drug-like* Space	Known Drug Space
Molecular weight (g mol^{-1})	0%	100%	100%
Lipophilicity (Log P)	20%	100%	100%
Hydrogen bond donors	100%	100%	100%
Hydrogen bond acceptors	0%	100%	100%
Polar surface area ($Å^2$)	0%	100%	100%
Rotatable bonds	0%	100%	100%
All criteria	0%	100%	100%

Table S14. 8-3′-Bicyclo[3.2.1]octanes studied within the defined chemical spaces.

Overall	*Lead-like* Space	*Drug-like* Space	Known Drug Space
Molecular weight (g mol^{-1})	0%	100%	100%
Lipophilicity (Log P)	10%	100%	100%
Hydrogen bond donors	100%	100%	100%
Hydrogen bond acceptors	0%	100%	100%
Polar surface area ($Å^2$)	0%	100%	100%
Rotatable bonds	0%	100%	100%
All criteria	0%	100%	100%

Table S15. Biphenyl ethers studied within the defined chemical spaces.

Overall	*Lead-like* Space	*Drug-like* Space	Known Drug Space
Molecular weight (g mol^{-1})	80%	100%	100%
Lipophilicity (Log P)	60%	100%	100%
Hydrogen bond donors	100%	100%	100%
Hydrogen bond acceptors	30%	100%	100%
Polar surface area ($Å^2$)	20%	100%	100%
Rotatable bonds	0%	100%	100%
All criteria	0%	100%	100%

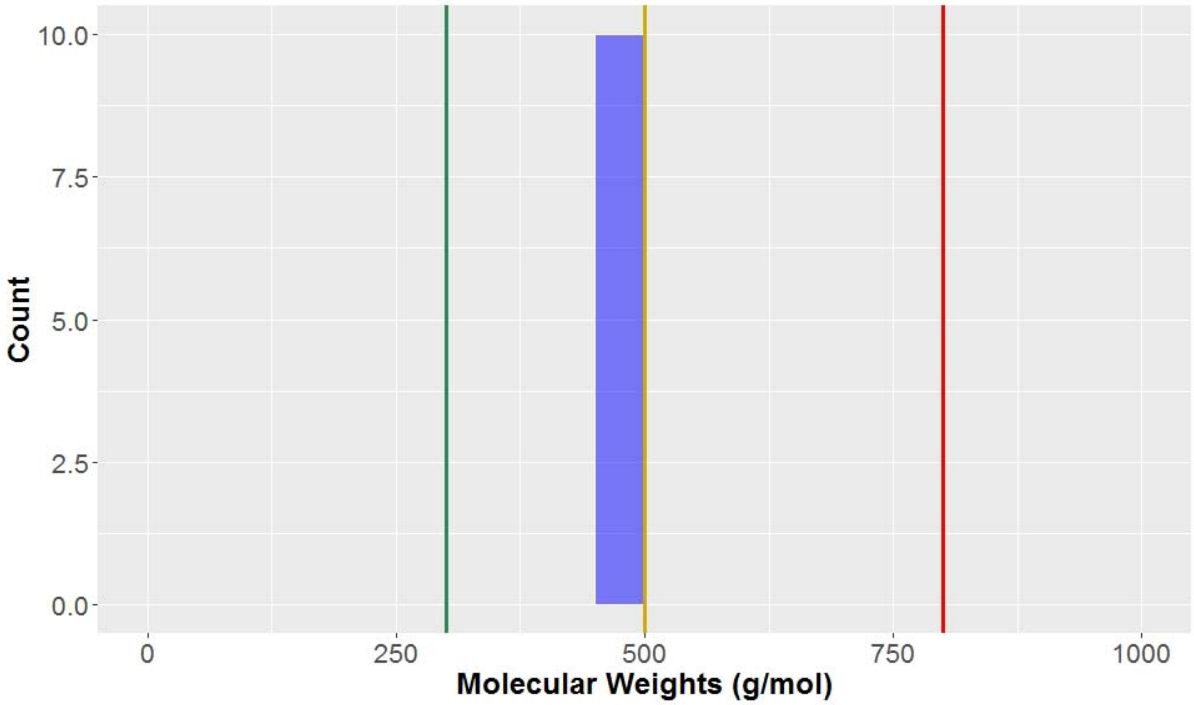

Figure S14. The statistical distribution of the molecular weight of the flavonolignans (green = 300 g mol^{-1}, compounds < 300 g $mol^{-1}$are in the *lead-like* space; yellow = 500 g mol^{-1}, compounds < 500 g mol^{-1} are in the *drug-like* space; red= 800 g mol^{-1}, compounds < 800 g $mol^{-1}$are in the KDS). Total number of compounds = 10.

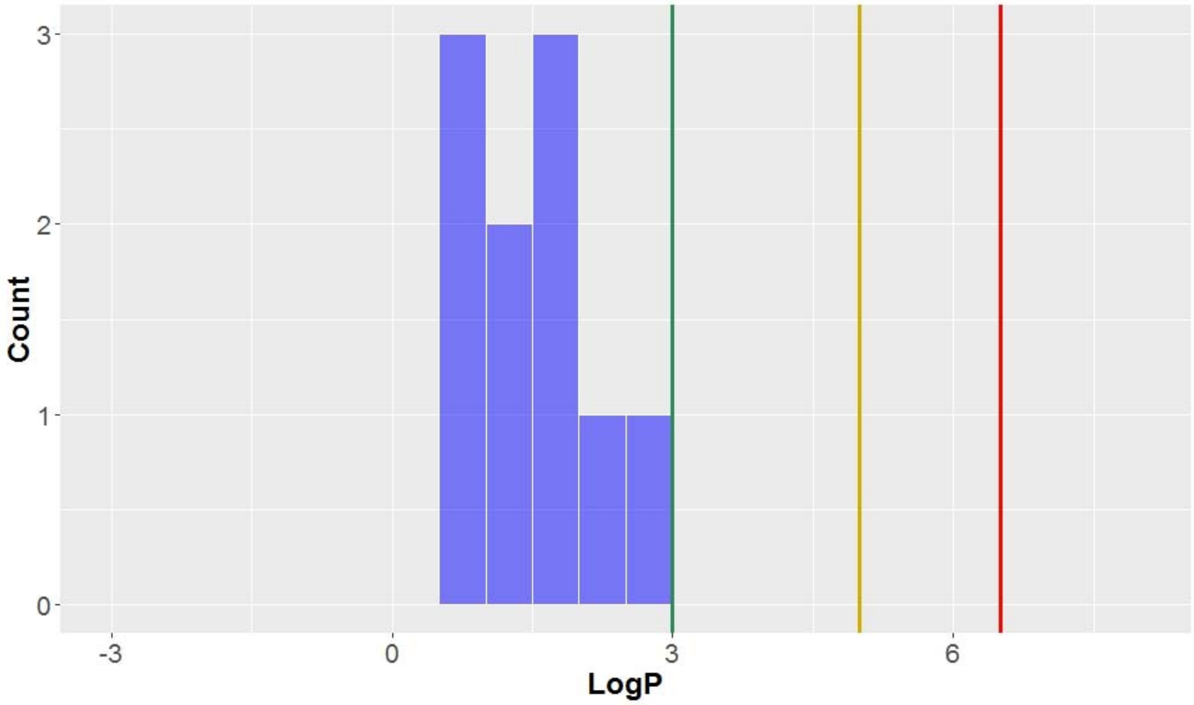

Figure S15. The statistical distribution of the octanol – water partition coefficient (LogP) of the flavonolignans (green = 3, compounds < 3 are in the *lead-like* space; yellow = 5, compounds < 5 are in the *drug-like* space; red= 6.5, compounds < 6.5 are in the KDS). Total number of compounds = 10.

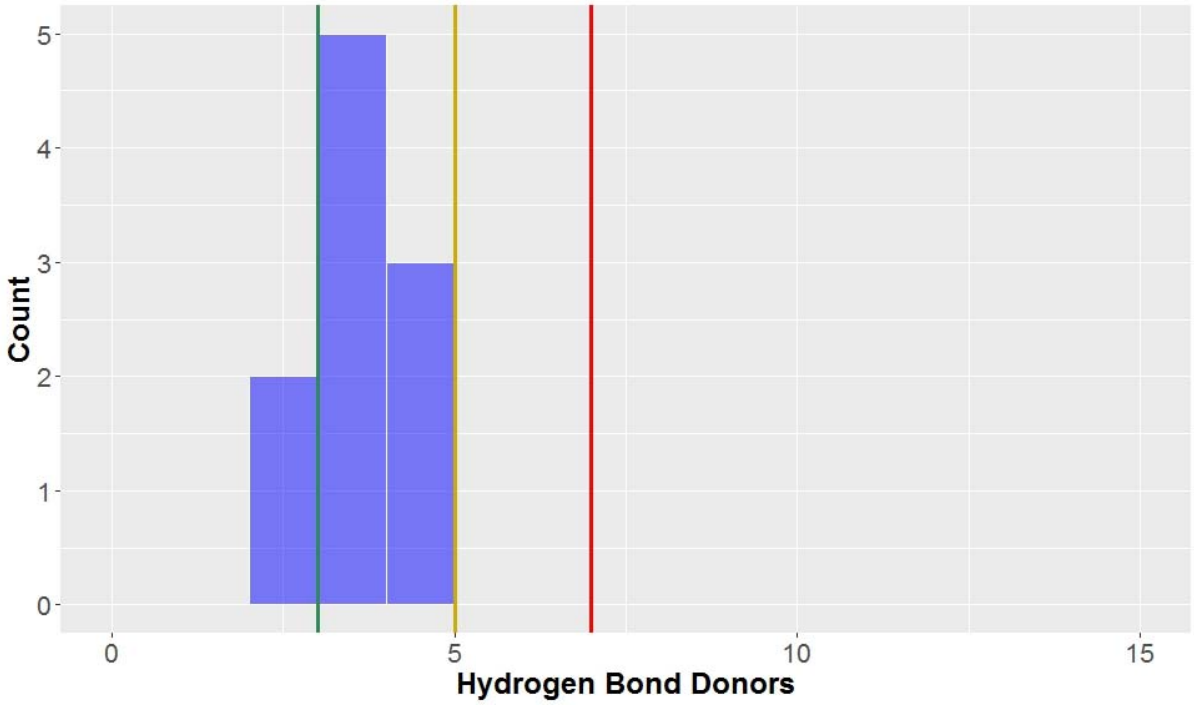

Figure S16. The statistical distribution of the hydrogen bond donors of flavonolignans (green = 3, compounds < 3 are in the *lead-like* space; yellow = 5, compounds < 5 are in the *drug-like* space; red= 7, compounds < 7 are in the KDS). Total number of compounds = 10.

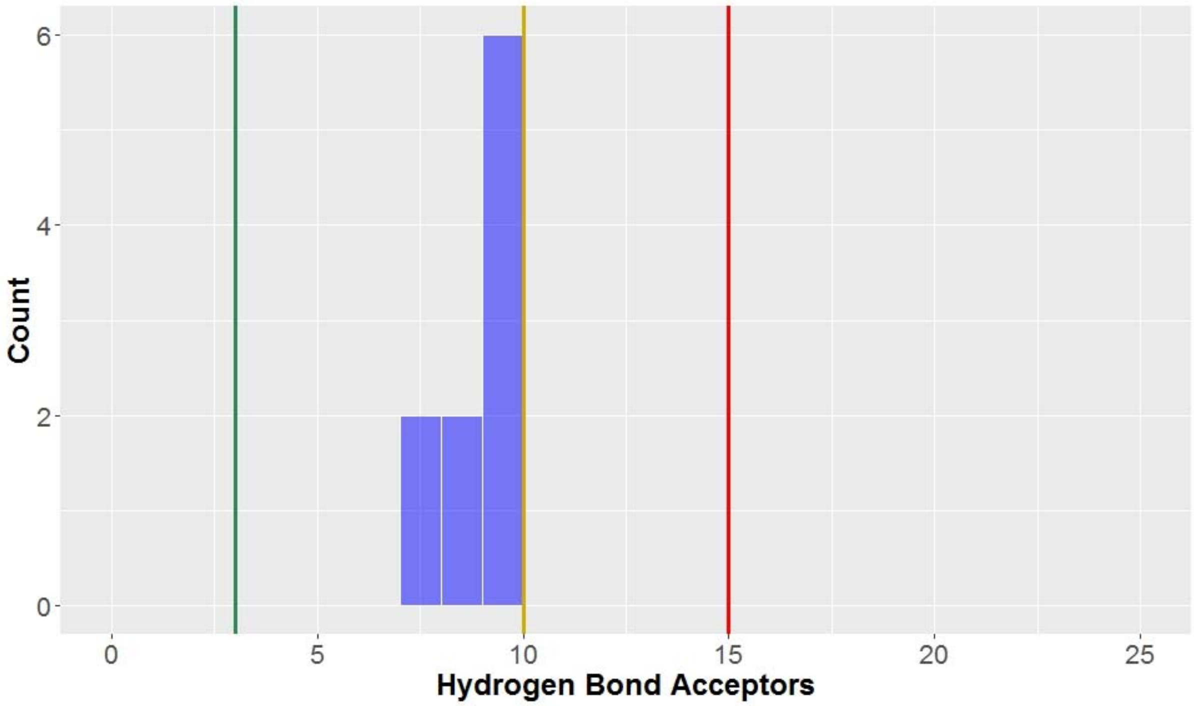

Figure S17. The statistical distribution of the hydrogen bond donors of flavonolignans (green = 3, compounds < 3 are in the *lead-like* space; yellow = 5, compounds < 5 are in the *drug-like* space; red= 7, compounds < 7 are in the KDS). Total number of compounds = 10.

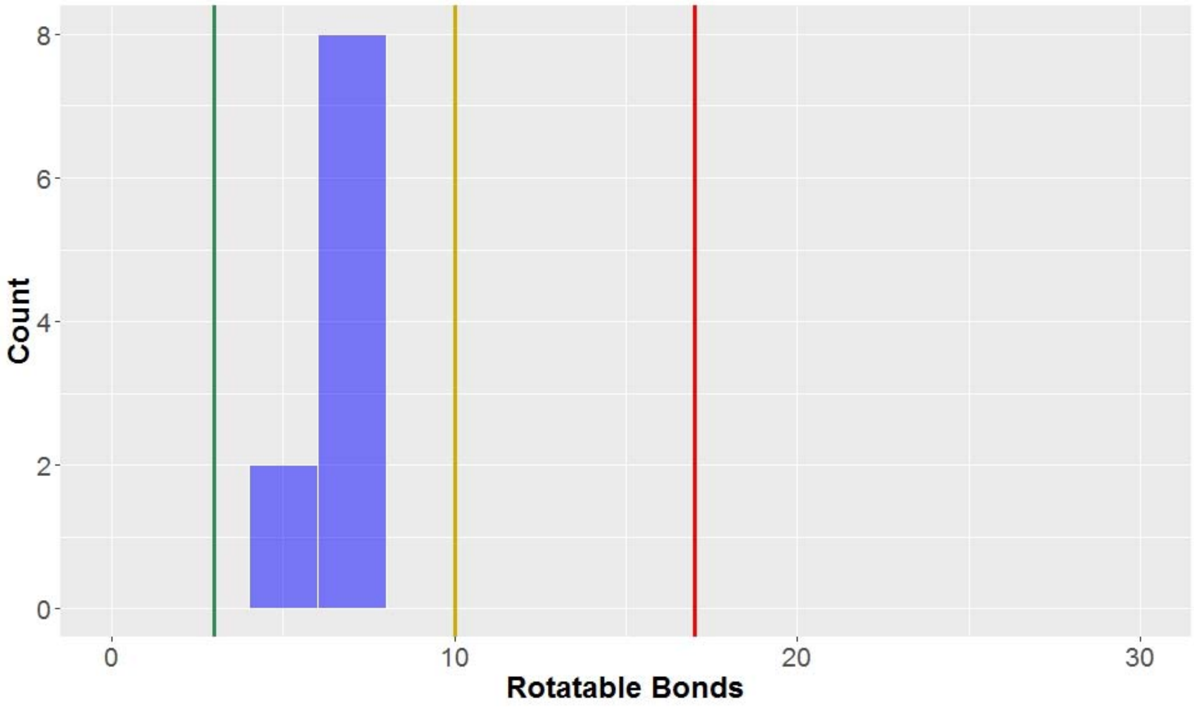

Figure S18. The statistical distribution of the rotatable bonds of the flavonolignans (green = 3, compounds < 3 are in the lead-like space; yellow = 10, compounds < 10 are in the drug-like space; red= 17, compounds < 17 are in the known drug space). Total number of compounds = 10.

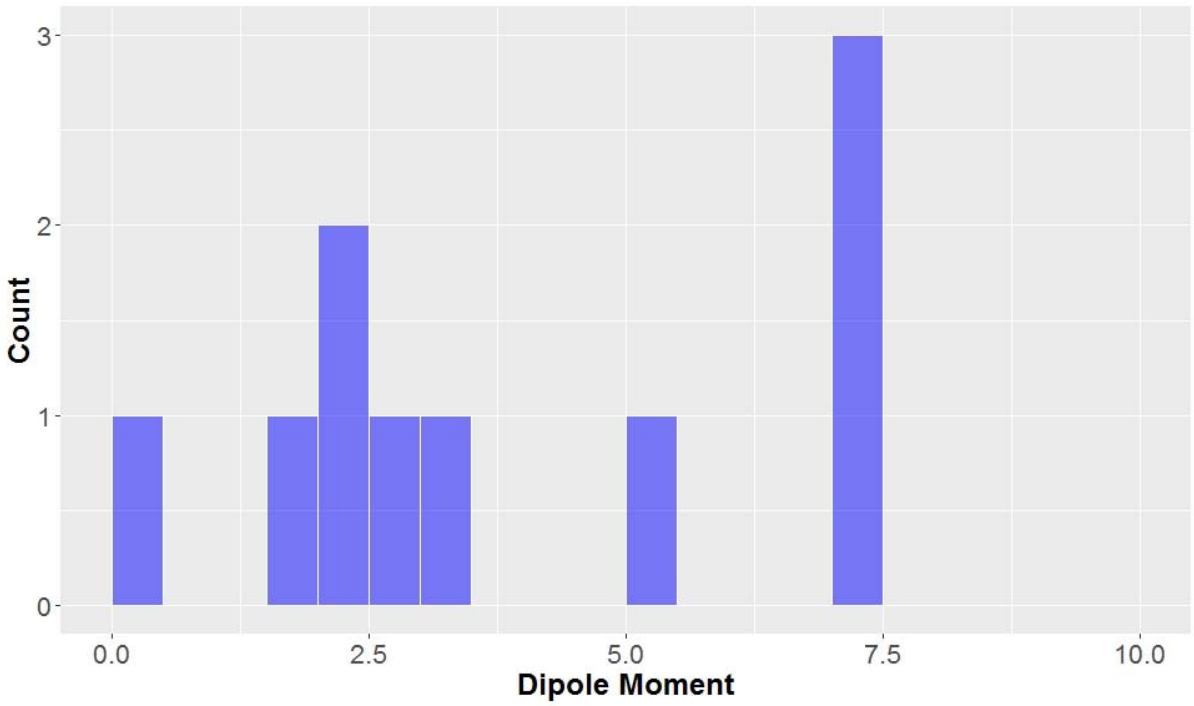

Figure S19. The statistical distribution of the dipole moment of the flavonolignans. Total number of compounds = 10.

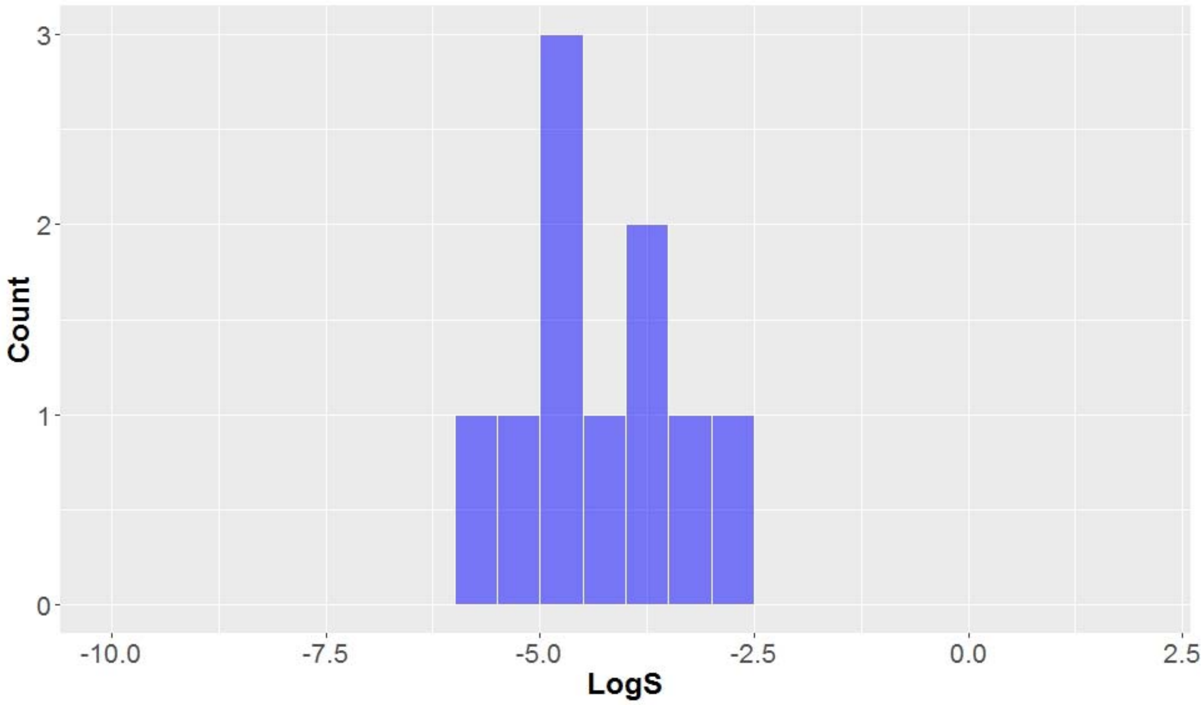

Figure S20. The statistical distribution of the water solubility (LogS) of the flavonolignans. Total number of compounds = 10.

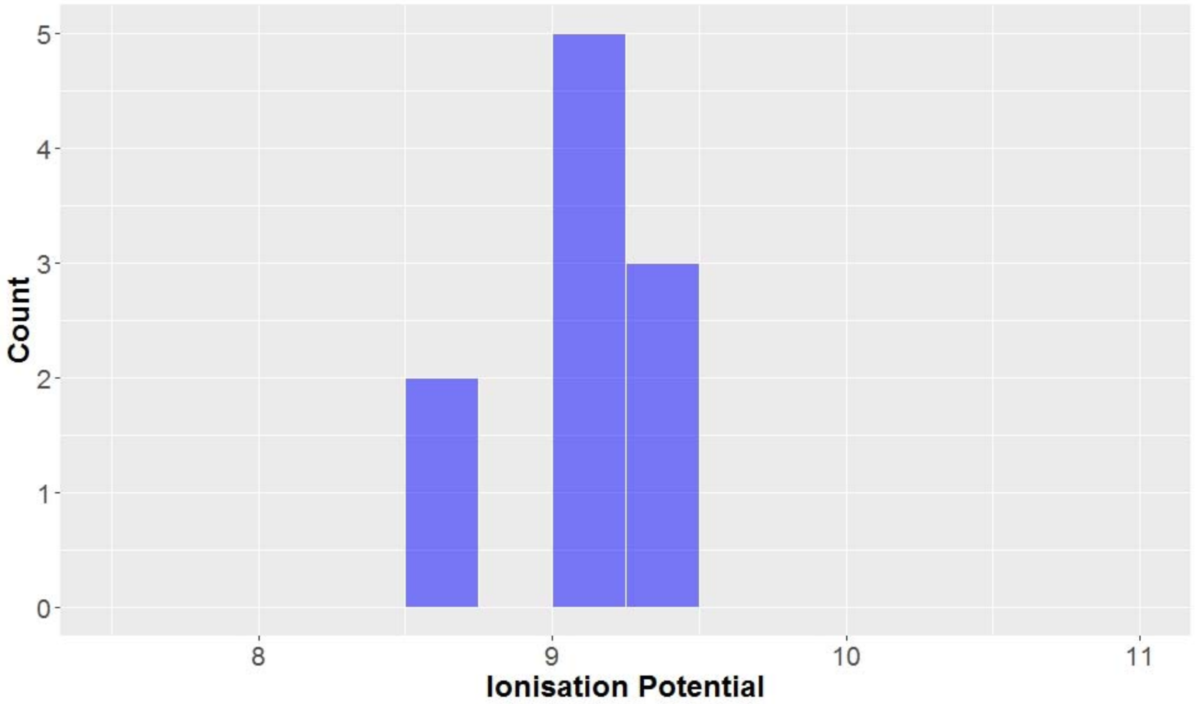

Figure S21. The statistical distribution of the ionisation potentials of the flavonolignans. Total number of compounds = 10.

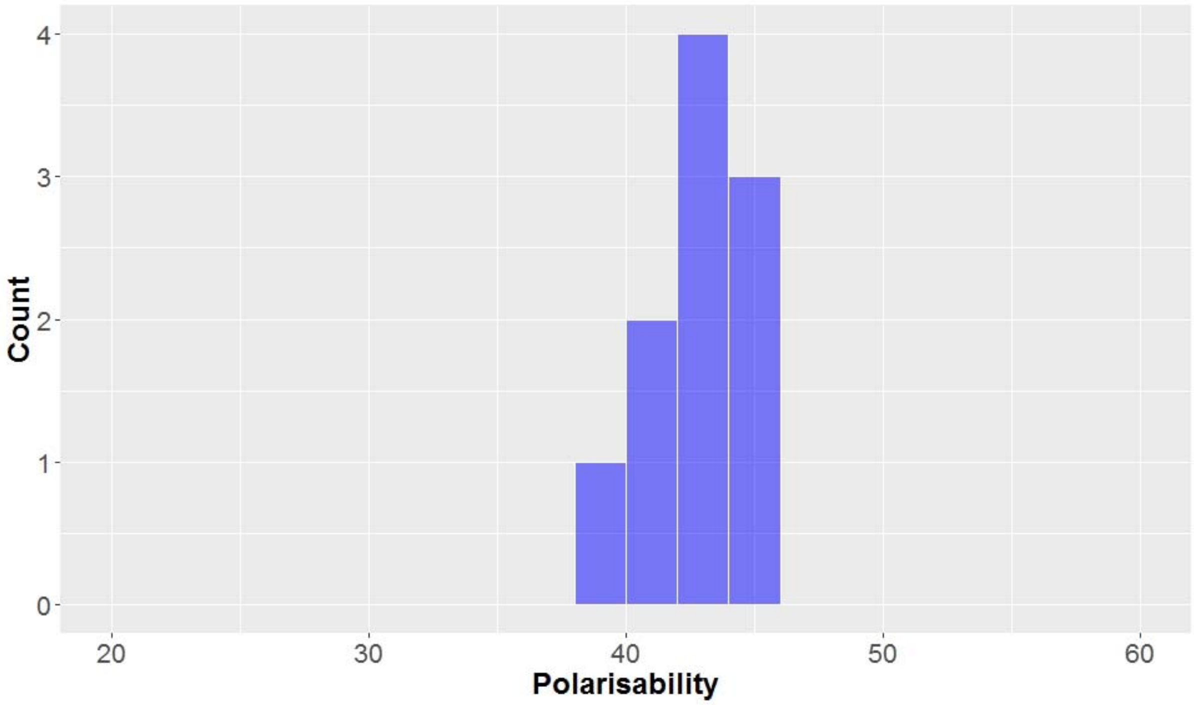

Figure S22. The statistical distribution of the polarisability of the flavonolignans. Total number of compounds = 10.

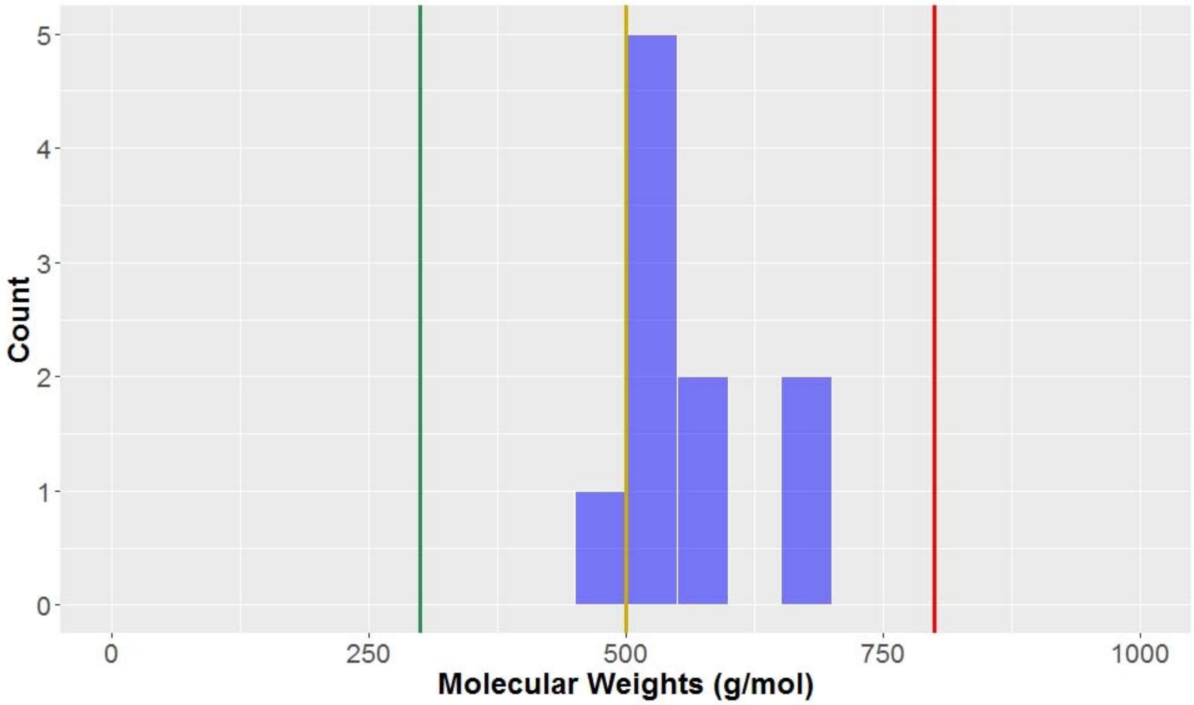

Figure S23. The statistical distribution of the molecular weight of the CLCs (green = 300 g mol^{-1}, compounds < 300 g $mol^{-1}$are in the *lead-like* space; yellow = 500 g mol^{-1}, compounds < 500 g mol^{-1} are in the *drug-like* space; red= 800 g mol^{-1}, compounds < 800 g $mol^{-1}$are in the KDS). Total number of compounds = 10.

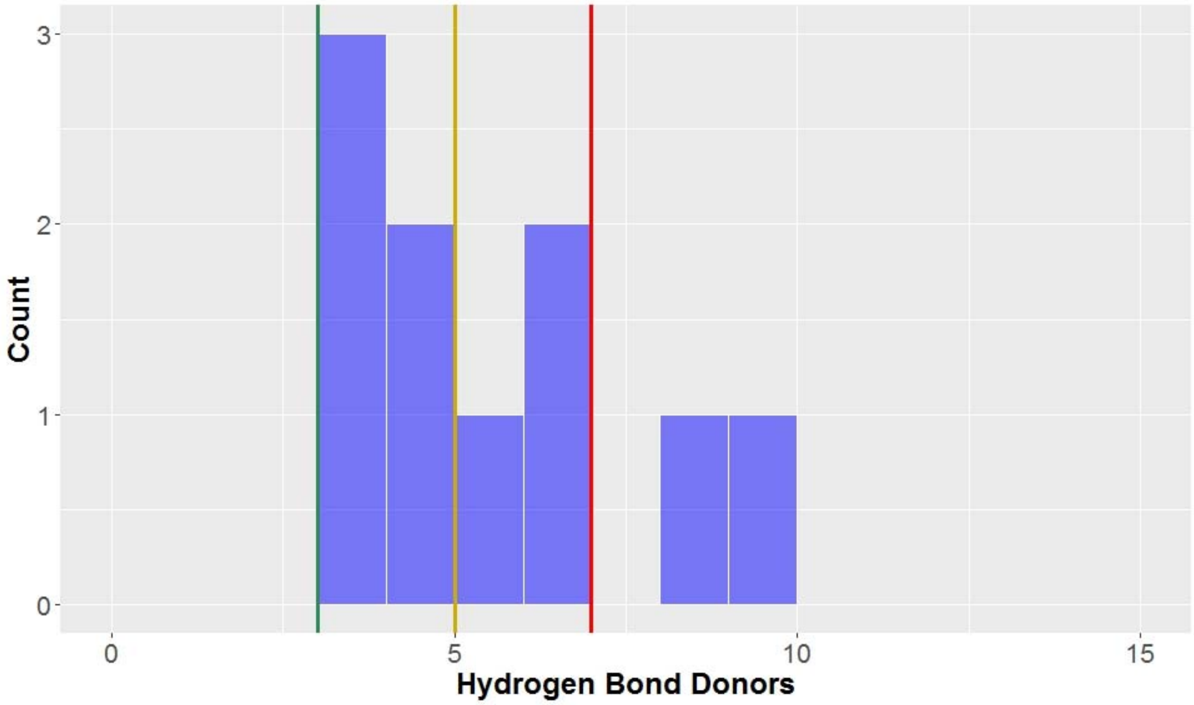

Figure S24. The statistical distribution of the hydrogen bond donors of the CLCs (green = 3, compounds < 3 are in the *lead-like* space; yellow = 5, compounds < 5 are in the *drug-like* space; red= 7, compounds < 7 are in the KDS). Total number of compounds = 10.

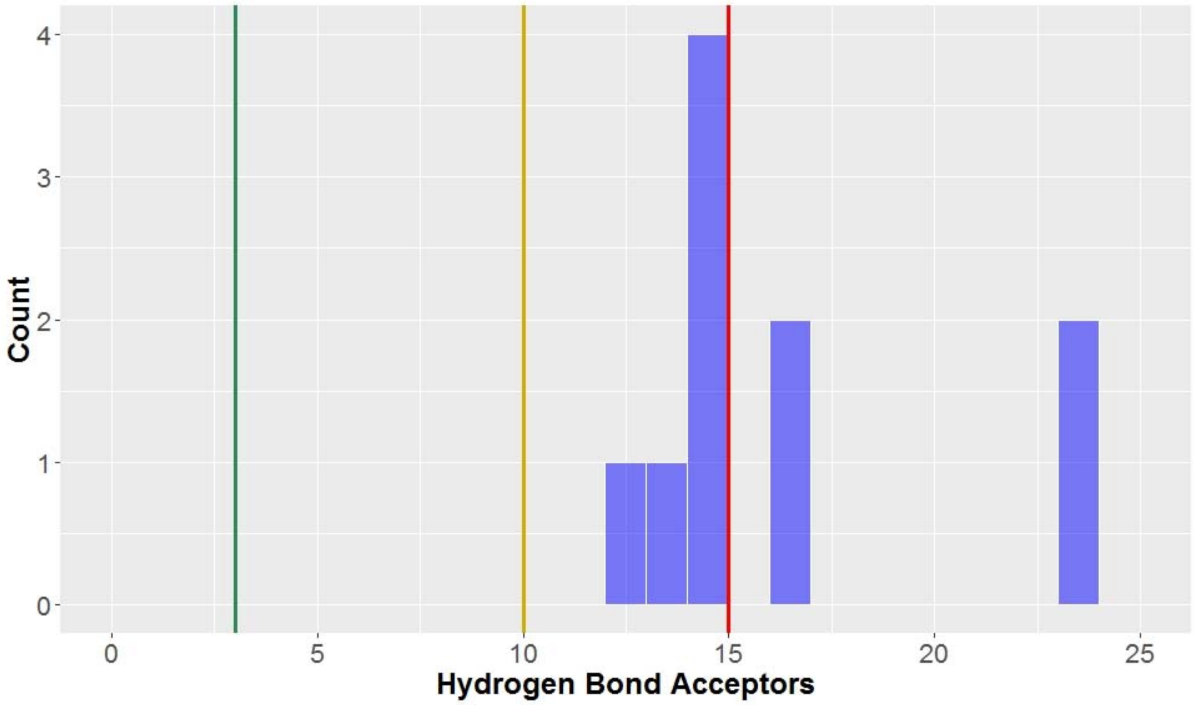

Figure S25. The statistical distribution of the hydrogen bond acceptors of the CLCs (green = 3, compounds < 3 are in the *lead-like* space; yellow = 5, compounds < 5 are in the *drug-like* space; red = 15, compounds < 15 are in the KDS). Total number of compounds = 10.

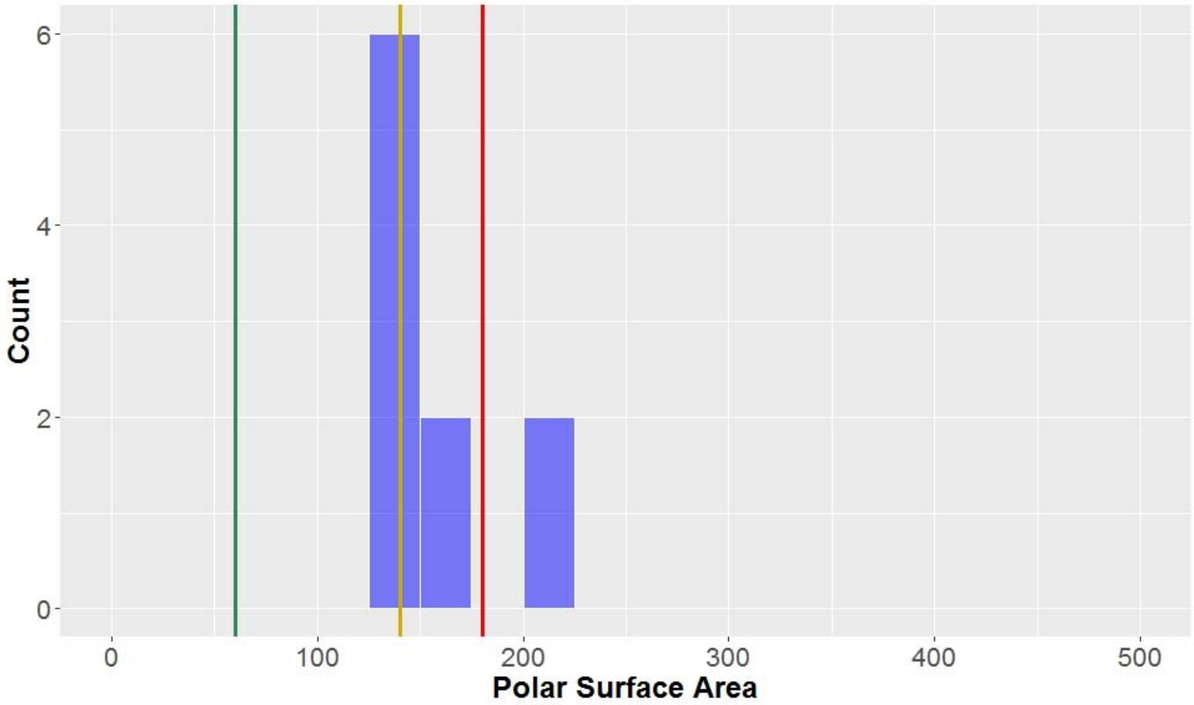

Figure S26. The statistical distribution of the polar surface area (PSA) of the sguars (green = 60, compounds < 60 Å^2 are in the *lead-like* space; yellow = 140, compounds < 140 Å^2 are in the *drug-like* space; red= 180, compounds < 180 Å^2 are in the KDS). Total number of compounds = 10.

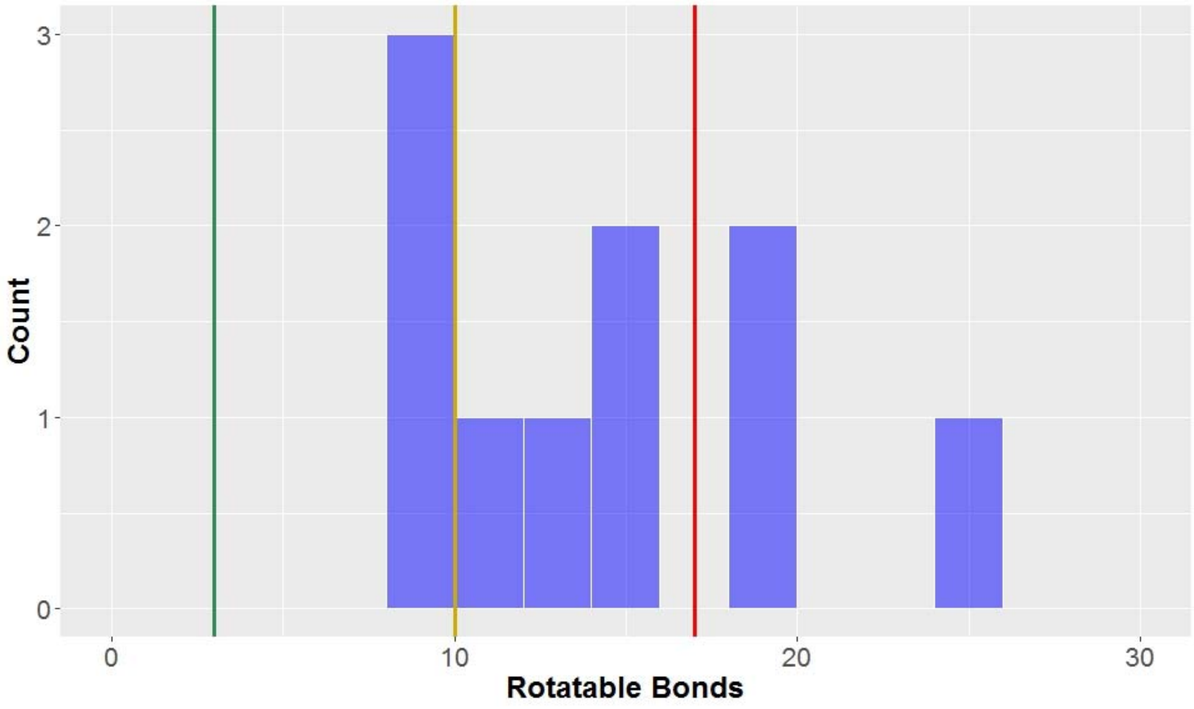

Figure S27. The statistical distribution of the rotatable bonds of the CLCs (green = 3, compounds < 3 are in the lead-like space; yellow = 10, compounds < 10 are in the drug-like space; red= 17, compounds < 17 are in the known drug space). Total number of compounds = 10.

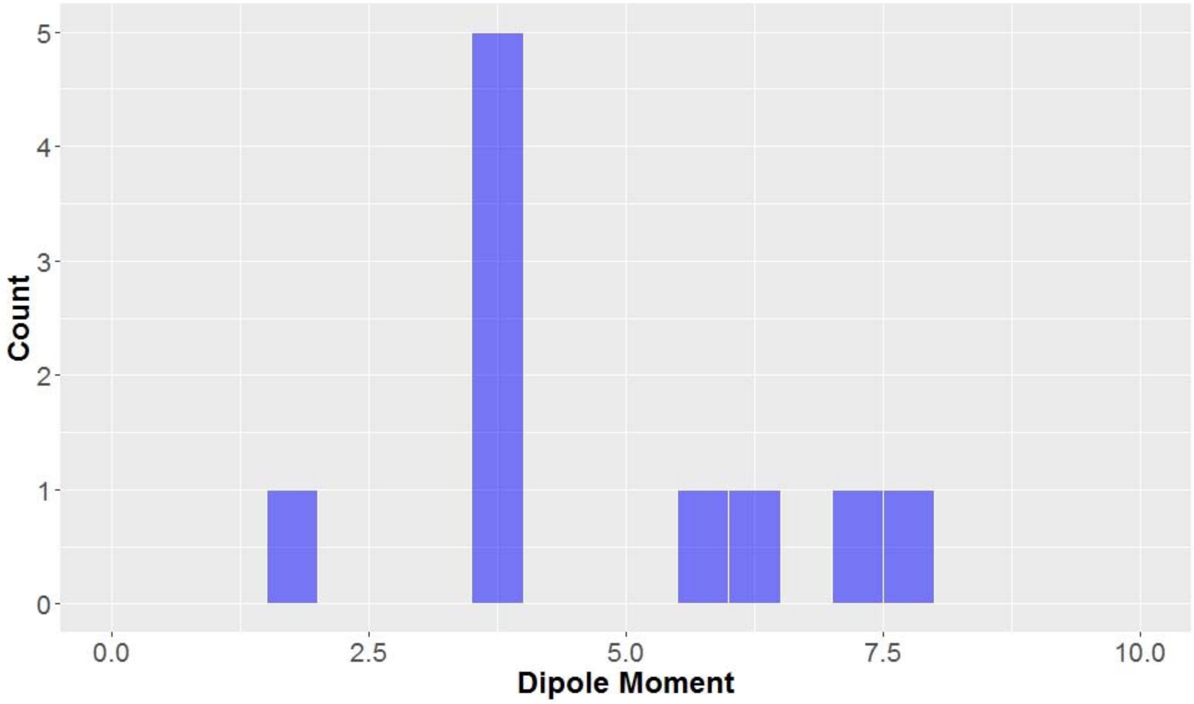

Figure S28. The statistical distribution of the dipole moments of the CLCs. Total number of compounds = 10.

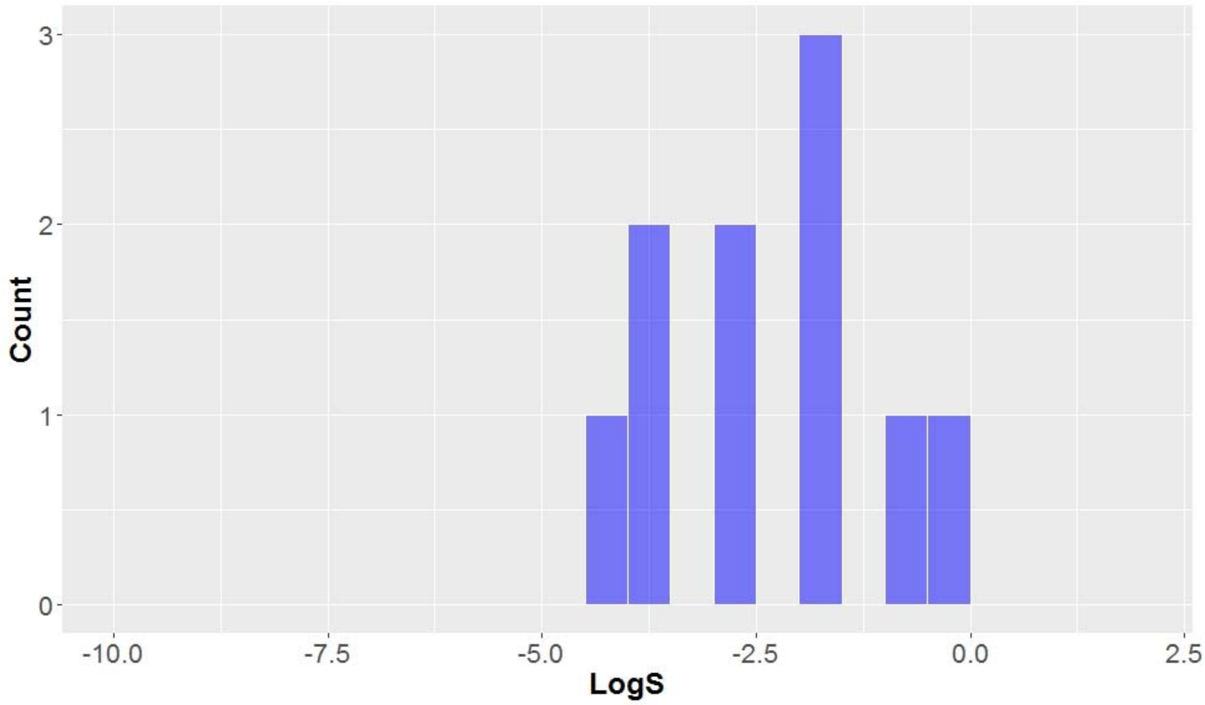

Figure S29. The statistical distribution of the water solubilities (LogS) of the CLCs. Total number of compounds = 10.

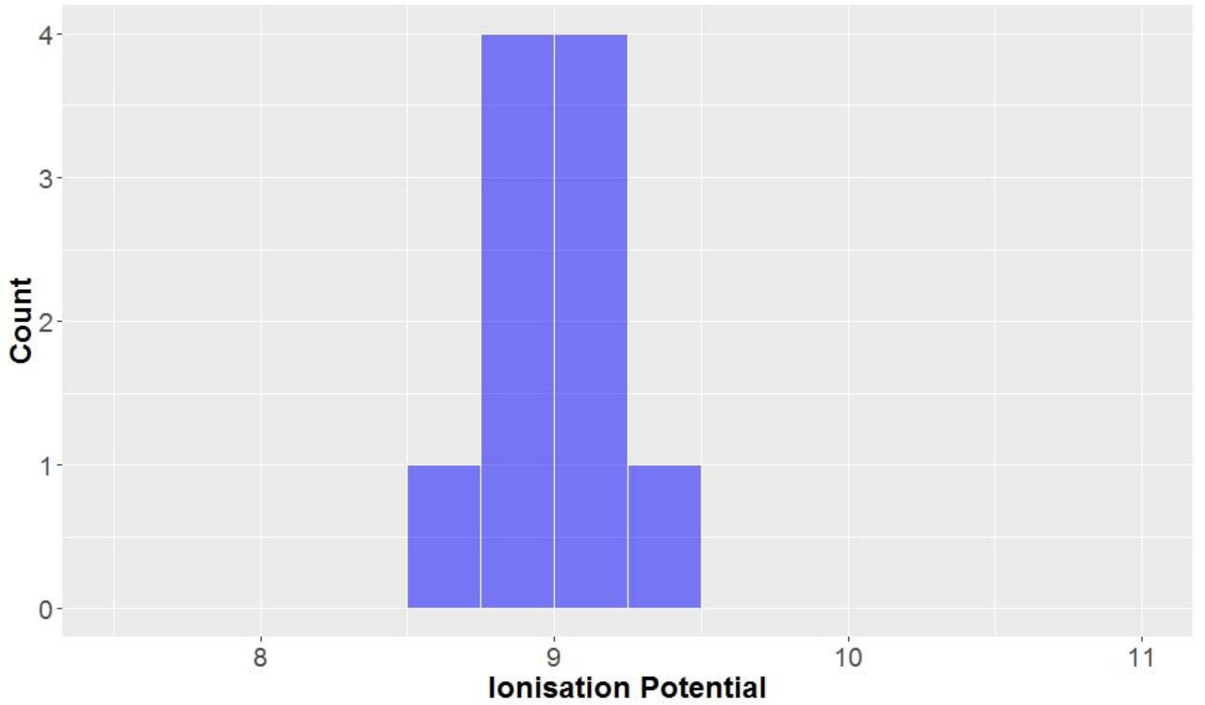

Figure S30. The statistical distribution of the ionisation potentials of the CLCs. Total number of compounds = 10.

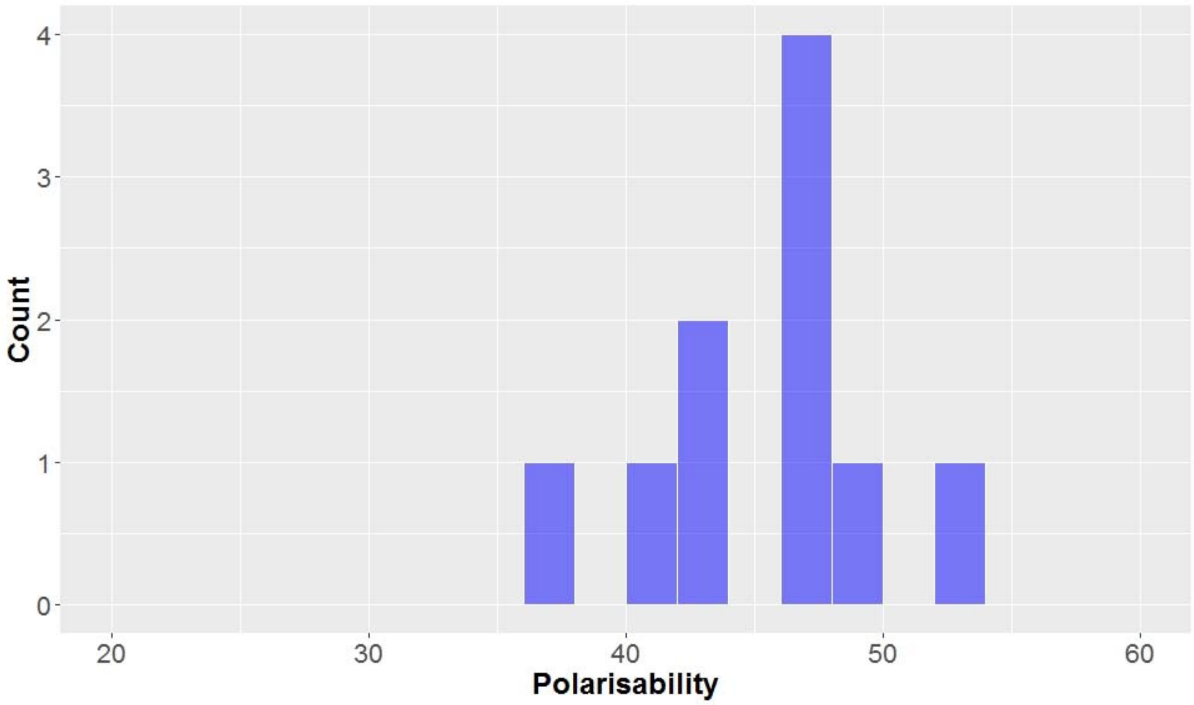

Figure S31. The statistical distribution of the polarisabilities of the CLCs. Total number of compounds = 10.

Table S16: The details, molecular descriptors and classification for the compounds used in this study.

Compound name	CAS	Molecular Weight	LogP	Hyrodgen Bond Donors	Hydrogen Bond Acceptors	Polar Surface Area	Rotatable Bonds	Dipole Moment	LogS	Ionisation Potential	Polaraisability	Type
Secoisolariciresinol	29388-59-8	362.422	2.175	4	6.4	97.366	13	2.601	-1.991	9.144	30.342	Dibenzylbutane
Enterodiol	80226-00-2	302.369	2.029	4	4.9	82.743	11	3.631	-2.785	9.409	29.258	Dibenzylbutane
Phyllanthin	10351-88-9	418.529	3.296	0	6.4	45.641	13	3.518	-6.156	9.025	39.812	Dibenzylbutane
Pregomisin	66280-26-0	390.475	4.534	2	4.5	71.391	11	4.608	-4.207	9.306	36.556	Dibenzylbutane
meso-Dihydroguaiaretic acid	66322-34-7	330.423	3.824	2	3	53.017	9	2.635	-3.182	8.913	29.499	Dibenzylbutane
(2R,3R)-2,3-Bis[(3,4-dimethoxyphenyl)methyl]-1,4-butanediol	58311-18-5	390.475	4.079	2	6.4	66.895	13	5.04	-3.983	9.011	36.901	Dibenzylbutane
Cinnamophilin	154677-96-0	344.407	2.875	2	5	75.951	9	3.264	-2.688	9.003	29.398	Dibenzylbutane
Demethyldihydroguaiaretic acid	71113-15-0	316.396	3.097	3	3	60.625	9	6.247	-2.571	8.968	27.366	Dibenzylbutane
rel-4-[(2R,3S)-4-(3,4-Dimethoxyphenyl)-2,3-dimethylbutyl]-2-methoxyphenol	171204-38-9	344.45	4.694	1	3	45.008	9	4.165	-3.794	8.758	32.664	Dibenzylbutane
3-Demethyl-(-)-secoisolariciresinol	151453-70-2	348.395	1.453	5	6.4	113.712	13	5.431	-2.479	9.228	30.458	Dibenzylbutane
(-)-Matairesinol	580-72-3	358.39	2.777	2	6	96.976	8	6.209	-3.764	8.911	34.317	Dibenzylbutyrolactone
(-)-Arctigenin	7770-78-7	372.417	2.884	1	6	80.919	8	3.847	-2.521	9.04	31.397	Dibenzylbutyrolactone
Enterolactone	78473-71-9	298.338	2.127	2	4.5	84.931	6	4.385	-2.946	9.663	28.759	Dibenzylbutyrolactone
(-)-Hydroxymatairesinol	20268-71-7	374.39	1.526	3	7.7	107.249	9	4.491	-2.052	9.307	29.927	Dibenzylbutyrolactone
(-)-Arcitin	25488-59-9	386.444	3.145	0	6	67.895	8	4.369	-1.802	8.948	32.913	Dibenzylbutyrolactone
Ketomatairesinol	53250-61-6	372.374	1.349	2	8	114.297	8	7.114	-1.651	9.248	29.064	Dibenzylbutyrolactone
(-)-3'-Desmethylarctigenin	147022-95-5	358.39	2.417	2	6	88.198	8	8.397	-2.581	9.274	30.312	Dibenzylbutyrolactone
(+)-Dimethylnortrachelogenin	33464-73-2	402.443	3.655	1	6.75	81.968	9	2.066	-3.257	9.366	36.542	Dibenzylbutyrolactone
(-)-Prestegane B	93376-04-6	358.39	2.493	2	6	87.685	8	4.817	-2.61	9.211	30.362	Dibenzylbutyrolactone
(-)-Podophyllotoxin	518-28-5	414.411	2.311	1	8.45	98.279	4	9.098	-2.78	9.004	35.985	Dibenzylbutyrolactone
Dehydroguaiaretic acid	20601-86-9	324.376	3.553	2	3	59.689	5	4.863	-4.276	8.362	31.868	Arylnapthalene

Sacidumlignan A	848986-16-3	384.428	3.832	2	4.5	71.437	7	3.52	-4.517	8.358	35.329	Arylnapthalene
Furfuracin A	1217184-44-5	324.376	3.638	2	3	55.595	5	4.573	-4.323	8.326	31.893	Arylnapthalene
Pycnanthulignene C	1207532-18-0	306.36	3.901	0	2.25	25.544	2	1.093	-5.489	8.25	31.628	Arylnapthalene
Pycnanthulignene D	1207532-19-1	352.386	4.024	1	3.75	47.495	4	0.633	-4.754	8.068	34.243	Arylnapthalene
Taiwanin C	14944-34-4	348.311	1.948	0	6	71.097	1	7.027	-1.892	8.386	29.841	Arylnapthalene
Chinensin	31888-76-3	364.354	2.578	0	6	69.288	3	7.869	-2.503	8.592	31.955	Arylnapthalene
Retrochinensin	5707-96-0	364.354	2.642	0	6	70.113	3	8.125	-2.476	8.84	32.328	Arylnapthalene
Justicidin B (7CI)	17951-19-8	364.354	2.645	0	6	69.529	3	5.012	-2.63	8.508	32.737	Arylnapthalene
Justicidin E	27792-97-8	348.311	1.806	0	6	75.877	1	8.364	-1.895	8.515	29.829	Arylnapthalene
(+)-Schizandrin	7432-28-2	446.539	5.377	1	5.25	55.63	7	2.799	-5.595	8.973	42.743	Dibenzocyclooctadien e
Schisandrin B	61281-37-6	400.471	3.517	0	4.5	40.552	4	3.274	-6.428	8.427	39.758	Dibenzocyclooctadien e
(+)-Schisandrin A	61281-38-7	416.513	3.624	0	4.5	39.031	6	2.246	-7.11	8.979	40.748	Dibenzocyclooctadien e
(+)-Gomisin A	58546-54-6	416.47	4.569	1	5.25	58.195	5	2.154	-5.143	8.618	39.387	Dibenzocyclooctadien e
(-)-Schisandrin C	61301-33-5	384.428	2.893	0	4.5	43.069	2	1.601	-5.139	8.234	36.295	Dibenzocyclooctadien e
(-)-Gomisin N	69176-52-9	400.471	3.515	0	4.5	40.518	4	3.247	-6.428	8.425	39.761	Dibenzocyclooctadien e
(-)-Gomisin J	66280-25-9	400.471	3.493	0	4.5	40.911	4	2.702	-6.393	8.496	39.682	Dibenzocyclooctadien e
Schisandrol A	58546-59-1	432.469	3.675	2	6.95	70.351	6	1.905	-4.338	8.526	38.774	Dibenzocyclooctadien e
Gomisin O	72960-22-6	416.47	4.2	1	6.2	55.882	5	1.48	-4.817	8.473	39.247	Dibenzocyclooctadien e
Gomisin H	66056-20-0	418.486	4.519	2	5.25	67.764	7	4.833	-4.784	9.166	39.095	Dibenzocyclooctadien e
(+)-Veraguensin	19950-55-1	372.46	4.395	0	4.7	38.914	4	3.05	-6.763	9.258	42.525	Substituted tetrahydrofuran
Nectandrin B	74683-16-2	344.407	3.482	2	4.7	66.802	4	4.742	-4.484	9.107	36.021	Substituted tetrahydrofuran
(±)-Galgravin	528-63-2	372.46	4.04	0	4.7	36.668	4	1.248	-6.398	9.001	41.224	Substituted tetrahydrofuran
(-)-Grandisin	53250-50-3	432.513	3.759	0	6.2	53.165	6	0.924	-6.711	9.385	45.201	Substituted tetrahydrofuran
(-)-Galbacin	528-64-3	340.375	2.75	0	4.7	42.44	0	2.127	-3.946	8.556	35.067	Substituted tetrahydrofuran
(+)-Verrucosin	83198-63-4	344.407	3.421	2	4.7	67.397	4	4.952	-4.37	9.073	35.7	Substituted tetrahydrofuran

(+)-Fragransin A2	112652-46-7	344.407	3.568	2	4.7	66.691	4	5.682	-5.039	9.065	36.995	Substituted tetrahydrofuran
(+)-Calopiptin	19950-67-5	356.418	3.083	0	4.7	40.962	2	4.489	-4.962	8.624	37.118	Substituted tetrahydrofuran
(-)-Galbelgin	10569-12-7	372.46	4.045	0	4.7	39.398	4	3.285	-6.364	8.92	41.082	Substituted tetrahydrofuran
Austrobailignin	55890-25-0	342.391	3.713	1	4.7	53.953	2	4.215	-4.692	8.611	35.915	Substituted tetrahydrofuran
(+)-Sesamin	607-80-7	354.359	1.712	0	6.4	52.084	0	0.303	-2.255	8.644	34.284	2,6-Diarylfurofuran
(+)-Pinoresinol	487-36-5	358.39	2.796	2	6.4	76.436	4	1.798	-4.314	9.163	36.208	2,6-Diarylfurofuran
(+)-Lirioresinol B	21453-69-0	418.443	3.163	2	7.9	86.669	6	3.687	-4.619	8.945	39.854	2,6-Diarylfurofuran
(+)-Asarinin	133-03-9	354.359	1.699	0	6.4	51.597	0	1.748	-2.244	8.622	34.239	2,6-Diarylfurofuran
(+)-Sesaminol	74061-79-3	370.358	2.445	1	7.15	70.978	1	1.831	-3.191	8.472	33.961	2,6-Diarylfurofuran
(+)-Mediaresinol	40957-99-1	388.416	2.955	2	7.15	82.906	5	3.806	-4.375	9.166	37.938	2,6-Diarylfurofuran
(+)-Phillygenin	487-39-8	372.417	3.56	1	6.4	62.257	4	3.465	-4.634	9.154	38.171	2,6-Diarylfurofuran
(+)-Epipinoresinol	24404-50-0	358.39	2.798	2	6.4	75.865	4	4.546	-4.356	9.116	36.231	2,6-Diarylfurofuran
(+)-Eudesmin	29106-36-3	386.444	2.91	0	6.4	48.464	4	2.058	-4.574	9.024	39.834	2,6-Diarylfurofuran
(+)-Magnolin	31008-18-1	416.47	2.913	0	7.15	55.647	5	3.495	-4.841	9.029	42.276	2,6-Diarylfurofuran
(7S,8R)-Lawsonicin	28199-69-1	360.406	2.641	3	6.4	86.485	9	3.583	-4.192	8.689	35.116	Benzofuran
(+)-Cedrusin	75775-36-9	346.379	1.802	4	6.4	98.75	9	4.481	-3.052	8.769	31.221	Benzofuran
5'-Methoxydehydroconiferyl alcohol	873077-50-0	390.432	2.844	3	7.15	93.542	10	2.695	-4.276	8.766	36.876	Benzofuran
3',4-O-Dimethylcedrusin	127179-41-3	374.433	3.4	2	6.4	75.055	9	3.291	-4.44	8.952	36.539	Benzofuran
Vladinol F	133318-48-6	360.406	2.586	3	6.4	86.264	9	3.435	-3.871	8.969	34.238	Benzofuran
(+)-Acuminatin	41744-39-2	340.418	4.605	0	3	30.634	4	2.597	-6.963	8.363	37.72	Benzofuran
(-)-Licarin A	51020-86-1	326.391	4.582	1	3	44.732	4	3.204	-5.746	8.246	35.704	Benzofuran
Dehydrodihydrodiisoeugeno l	4731-87-7	328.407	4.58	1	3	44.664	5	2.965	-5.162	8.804	34.951	Benzofuran
Dehydrodiconiferyl alcohol	4263-87-0	358.39	2.53	3	6.4	87.27	8	1.097	-4.249	8.571	35.145	Benzofuran
Dehydrodiisoeugenol	2680-81-1	326.391	4.486	1	3	44.724	4	2.863	-5.169	8.401	35.064	Benzofuran
1,4-Benzodioxin-6-propanol, 2,3-dihydro-3-(4-hydroxy-3-methoxyphenyl)-2-(hydroxymethyl)-, (2S,3S)-	144881-21-0	346.379	2.322	3	6.4	85.05	8	3.613	-3.732	9.215	33.4	Benzodioxane

(±)-Isoamericanol A	133838-66-1	330.337	1.322	4	6.4	99.602	7	6.031	-3.135	8.888	30.757	Benzodioxane
(-)-Eusiderin A	59332-00-2	386.444	4.308	0	4.5	45.838	6	2.828	-6.868	9.066	40.859	Benzodioxane
Isoamericanin A	109063-85-6	330.337	1.43	3	6.7	115.439	7	2.513	-3.44	9.338	31.795	Benzodioxane
(-)-Eusiderin C	76333-70-5	386.444	3.543	0	4.5	45.886	6	4.046	-6.191	9.124	38.014	Benzodioxane
(±)-Eusiderin E	97730-86-4	372.417	4.664	1	4.5	59.485	5	4.77	-5.783	8.742	39.014	Benzodioxane
(±)-Eusiderin G	101508-18-3	400.427	3.688	0	6.5	82.9	7	8.018	-4.549	9.127	39.513	Benzodioxane
(±)-Eusiderin K	126176-81-6	372.417	4.669	1	4.5	59.115	6	3.991	-5.578	9.043	38.401	Benzodioxane
cis-Rogersinine A	666250-51-7	298.338	3.441	2	3	56.658	3	1.418	-4.648	8.698	32.415	Benzodioxane
cis-Rogersinine B	666250-53-9	296.322	3.417	2	3	56.626	3	1.528	-4.755	8.935	32.351	Benzodioxane
Myrislignan	171485-39-5	374.433	3.688	2	5.45	63.165	11	4.868	-3.207	8.858	33.305	Alkyl aryl ether
1,3-Propanediol, 1-(4-hydroxy-3-methoxyphenyl)-2-[4-[(1E)-3-hydroxy-1-propen-1-yl]-2-methoxyphenoxy]-, (1R,2R)-rel-	126061-41-4	376.405	1.692	4	8.1	104.167	13	2.326	-2.522	9.21	32.39	Alkyl aryl ether
Benzenemethanol, α-[(1R)-1-[2,6-dimethoxy-4-(2-propen-1-yl)phenoxy]ethyl]-3,4-dimethoxy-, (αS)-rel-	93289-62-4	388.46	4.967	1	5.45	48.489	11	5.715	-5.054	9.061	38.79	Alkyl aryl ether
1,3-Propanediol, 1-(4-hydroxy-3-methoxyphenyl)-2-[4-(3-hydroxypropyl)-2-methoxyphenoxy]-, (1R,2R)-	97133-59-0	378.421	1.825	4	8.1	101.378	14	6.181	-2.542	9.074	32.664	Alkyl aryl ether
1,3-Propanediol, 1-(4-hydroxy-3-methoxyphenyl)-2-[4-[(1E)-3-hydroxy-1-propen-1-yl]-2-methoxyphenoxy]-, (1R,2S)-rel-	126107-59-3	376.405	1.946	4	8.1	98.663	13	4.3	-3.138	9.012	34.061	Alkyl aryl ether
Virolongin A	94608-22-7	402.486	4.821	0	4.5	43.532	10	4.526	-7.885	8.823	41.923	Alkyl aryl ether
1,3-Propanediol, 1-(4-hydroxy-3-methoxyphenyl)-2-[4-[(1E)-3-hydroxy-1-propen-1-yl]-2-methoxyphenoxy]-, (1R,2S)-	168252-52-6	376.405	1.689	4	8.1	104.164	13	2.281	-2.731	9.122	32.763	Alkyl aryl ether
Rhaphidecursinol B	52190-20-2	418.486	4.987	1	6.2	61.757	12	4.828	-4.756	9.289	40.44	Alkyl aryl ether

Virolongin B	124151-41-3	402.486	3.459	0	4.5	40.038	11	1.796	-6.706	8.962	36.422	Alkyl aryl ether
1,3-Propanediol, 1-(4-hydroxy-3-methoxyphenyl)-2-[4-[(1E)-3-hydroxy-1-propen-1-yl]-2-methoxyphenoxy]-, (1S,2R)-	890317-92-7	376.405	1.875	4	8.1	103.587	13	3.428	-3.122	9.126	34.025	Alkyl aryl ether
Magnolignan	20601-85-8	270.371	4.963	2	1.5	37.612	7	3.409	-4.228	8.829	30.244	Biphenyl
Tetrahydrohonokiol	35406-31-6	270.371	4.983	2	1.5	38.686	7	0.983	-4.195	8.772	29.516	Biphenyl
Honokiol	35354-74-6	266.339	4.992	2	1.5	37.771	7	1.017	-4.123	8.791	29.354	Biphenyl
Magnolol	528-43-8	266.339	4.991	2	1.5	37.593	7	3.212	-4.08	8.846	29.128	Biphenyl
Biseugenol A	1807921-16-9	326.391	4.478	2	3	50.462	9	3.547	-4.767	8.578	33.86	Biphenyl
Neglingnan C	1441710-14-0	414.411	2.117	0	8.5	91.926	7	3.523	-2.42	8.489	36.234	Biphenyl
Neglingnan D	1441710-21-9	418.443	2.799	2	8.5	99.997	11	6.121	-2.999	9.002	35.417	Biphenyl
Streblusol D	1399052-36-8	334.368	0.587	6	8.3	111.551	13	5.784	-1.913	8.776	28.368	Biphenyl
erythro-Streblusol B	1399052-32-4	328.407	3.316	3	4.9	64.147	10	4.068	-3.987	8.692	33.433	Biphenyl
Streblusol E	1399052-37-9	242.274	2.235	3	2.25	62	6	4.46	-2.77	8.593	24.438	Biphenyl
Isotruxillic acid	528-34-7	296.322	3.379	2	4	89.629	2	6.946	-3.843	10.177	31.739	Cyclobutane
Dimethyl β-truxinate	36650-44-9	324.376	4.214	0	4	61.618	2	2.879	-5.26	9.86	38.276	Cyclobutane
Magnosalin	81861-74-7	416.513	4.189	0	4.5	41.156	6	0.962	-7.641	8.774	43.133	Cyclobutane
Andamanicin	130323-08-9	416.513	4.383	0	4.5	40.923	6	5.091	-7.854	8.811	43.865	Cyclobutane
Endiandrin A	946065-33-4	328.407	4.035	2	3	59.323	4	3.945	-5.082	9.008	35.449	Cyclobutane
Heterotropan	70280-35-2	416.513	3.581	0	4.5	38.295	6	2.765	-7.251	8.535	41.545	Cyclobutane
Cinbalansan	58045-93-5	356.461	3.753	0	3	32.06	4	4.589	-6.929	9.189	37.217	Cyclobutane
Dimethyl 3,4,3',4'-tetrahydroxy-δ-truxinate	1383572-08-4	388.373	1.478	4	7	152.012	6	3.821	-4.553	9.139	37.933	Cyclobutane
Endiandrin B	1140478-56-3	328.407	3.668	2	3	59.85	4	4.791	-3.821	9.074	32.653	Cyclobutane
3,3',4,4'-Tetrahydroxy-β-truxinic acid	128009-22-3	444.48	4.333	0	7	92.618	6	1.166	-5.486	8.896	44.986	Cyclobutane
3a,6-Methano-3aH-cyclohepta-1,3-dioxol-7(4H)-one, 5,6-dihydro-4-methyl-6-(2-propen-1-yl)-5-(3,4,5-trimethoxyphenyl)-, (3aS,4S,5R,6S)-	1651214-81-1	386.444	3.831	0	5.75	63.309	5	4.472	-3.842	9.441	37.262	8-1′-Bicyclo[3.2.1]octane

3a,6-Methano-3aH-cyclohepta-1,3-dioxol-7(4H)-one, 5,6-dihydro-4-methyl-6-(2-propen-1-yl)-5-(3,4,5-trimethoxyphenyl)-, (3aR,4R,5R,6R)-	1651214-82-2	386.444	3.9	0	5.75	61.303	5	4.705	-3.556	9.682	37.014	8-1′-Bicyclo[3.2.1]octane
(+)-Ocobullenone	149990-50-1	370.401	3.087	0	5.75	62.485	3	5.044	-2.745	8.478	33.435	8-1′-Bicyclo[3.2.1]octane
Sibyllenone	299175-09-0	370.401	3.114	0	5.75	66.05	3	4.68	-3.113	8.624	34.196	8-1′-Bicyclo[3.2.1]octane
Isoocobullenone	165306-72-9	370.401	3.258	0	5.75	62.242	3	3.764	-3.165	8.598	34.53	8-1′-Bicyclo[3.2.1]octane
Kadsurenin D	140669-89-2	356.418	3.211	0	6.25	68.599	5	5.183	-3.791	9.15	36.842	8-1′-Bicyclo[3.2.1]octane
Kadsurenin H	140669-88-1	400.471	4.21	0	6.25	82.085	6	7.051	-4.493	9.303	41.989	8-1′-Bicyclo[3.2.1]octane
Canellin A	54835-74-4	376.449	2.91	2	7.35	66.969	6	4.092	-3.542	8.393	35.354	8-1′-Bicyclo[3.2.1]octane
Kadsurenin J	145553-02-2	400.471	3.801	0	6.25	81.38	6	7.405	-3.916	8.893	39.44	8-1′-Bicyclo[3.2.1]octane
Canellin A	54835-72-2	360.406	2.148	2	7.65	80.862	5	5.708	-3.09	8.464	33.388	8-1′-Bicyclo[3.2.1]octane
Ocophyllol A	1189119-90-1	342.391	3.082	1	5.95	65.468	4	3.683	-3.758	8.707	33.849	8-3′-Bicyclo[3.2.1]octane
Ocophyllol B	1189119-91-2	358.433	3.743	1	5.95	64.04	6	2.434	-4.446	9.444	37.017	8-3′-Bicyclo[3.2.1]octane
Kadsurenin L	149438-61-9	400.471	3.999	0	6.25	82.229	6	5.333	-4.849	10.014	41.341	8-3′-Bicyclo[3.2.1]octane
Macrophyllin B	74944-98-2	358.433	3.831	1	5.95	60.516	6	4.433	-4.642	9.492	37.024	8-3′-Bicyclo[3.2.1]octane
Nectamazin A	1187947-44-9	418.486	4.034	1	7.45	73.648	8	7.788	-4.587	9.141	40.554	8-3′-Bicyclo[3.2.1]octane
Nectamazin B	1187947-50-7	418.486	4.181	1	7.45	72.224	8	5.72	-4.947	9.737	41.601	8-3′-Bicyclo[3.2.1]octane
Kadsurenin B	145701-13-9	342.391	3.039	1	5.95	66.976	4	4.569	-3.611	8.503	33.491	8-3′-Bicyclo[3.2.1]octane
Cinerin D	1166328-51-3	402.443	3.467	1	7.45	74.228	6	2.193	-4.135	8.495	38.52	8-3′-Bicyclo[3.2.1]octane
Cinerin A	1166328-44-4	400.427	2.626	0	7.75	83.365	5	5.849	-2.593	8.718	36.757	8-3′-Bicyclo[3.2.1]octane
Cinerin B	1166328-46-6	372.417	3.072	1	6.7	74.982	5	2.868	-3.685	8.53	34.955	8-3′-Bicyclo[3.2.1]octane
Obovatol	83864-78-2	282.338	4.557	2	2	48.41	8	2.887	-4.035	9.186	29.813	Biphenyl ether
Aristogin A	101110-74-1	286.284	2.074	0	5.25	84.565	5	7.249	-2.903	9.895	29.411	Biphenyl ether
Aristogin E	113275-15-3	288.299	2.612	1	4.95	71.306	6	4.799	-3.579	9.663	29.474	Biphenyl ether

Aristogin B	135303-87-6	286.284	2.076	0	5.25	84.618	5	5.751	-2.914	9.918	29.42	Biphenyl ether
Tetrahydroobovatol	83864-79-3	286.37	4.566	2	2	48.386	8	2.873	-4.254	9.167	30.198	Biphenyl ether
Obovatal	83864-77-1	296.322	2.591	2	4	86.014	9	5.817	-3.215	9.248	28.598	Biphenyl ether
3-Methylobovatol	122738-75-4	296.365	4.919	1	2	34.479	8	3.992	-4.473	9.345	31.626	Biphenyl ether
Obovaaldehyde	83864-76-0	270.284	2.206	2	4	84.235	7	4.711	-2.908	9.615	26.379	Biphenyl ether
2-Propenoic acid, 3,3'-[oxybis(3-methoxy-4,1-phenylene)]bis-, (2E,2'E)-	300849-01-8	370.358	3.31	2	6	122.037	10	9.998	-3.804	9.698	33.684	Biphenyl ether
Aristogin D	477199-86-3	302.283	2.289	1	5.25	97.382	5	4.913	-3.83	9.921	30.726	Biphenyl ether
Silybin A	22888-70-6	482.443	1.569	4	9.65	160.376	7	3.376	-4.614	9.268	44.074	Flavonolignan
(+)-Silychristin	33889-69-9	482.443	0.951	5	9.65	175.055	8	1.825	-3.992	9.068	41.977	Flavonolignan
Silybin B	142797-34-0	482.443	1.455	4	9.65	157.946	7	2.287	-3.871	9.249	42.093	Flavonolignan
Isosilybin B	142796-22-3	482.443	1.297	4	9.65	161.924	7	0.368	-3.454	9.295	41.181	Flavonolignan
2,3-Dehydrosilybin	25166-14-7	480.427	1.964	4	8.45	156.678	7	7.164	-4.984	8.681	43.839	Flavonolignan
Isosilychristin	77182-66-2	482.443	0.893	5	9.65	163.105	8	5.166	-2.943	9.101	38.839	Flavonolignan
(-)-Silandrin	70815-32-6	466.443	2.427	3	7.95	141.679	6	2.97	-5.23	9.329	44.049	Flavonolignan
Rhodiolin	86831-53-0	480.427	1.994	4	8.45	151.818	7	7.117	-4.985	8.712	43.797	Flavonolignan
(-)-Silychristin B	879325-58-3	482.443	0.995	5	9.65	174.372	8	2.364	-4.269	9.077	42.65	Flavonolignan
Hydnocarpin	51419-48-8	464.428	2.538	3	7.7	141.727	6	7.009	-5.52	9.155	44.706	Flavonolignan
(-)-Secoisolariciresinol 9'-O-β-D-glucopyranoside	63320-67-2	524.564	0.064	7	14.9	150.678	19	6.413	-0.87	9.133	36.777	Sugar
Arctigenin-4-glucoside	20362-31-6	534.559	0.975	4	14.5	137.659	14	5.647	-1.994	9.156	41.9	Sugar
(+)-Lyoniresinol 9'-O-β-glucoside	87585-32-8	582.6	0.304	7	16.4	172.444	16	3.775	-2.895	8.906	47.611	Sugar
Schisandroside C	2059120-56-6	534.559	1.617	5	13	141.487	10	7.05	-3.83	8.679	46.407	Sugar
Clemastanin B	112747-98-5	684.69	-1.383	9	23.4	211.672	20	3.989	-1.959	9.321	53.851	Sugar
Phillyroside	487-41-2	534.559	1.537	4	14.9	126.758	10	1.72	-3.833	8.983	48.415	Sugar
Cedrusin 4-O-β-glucoside	131723-83-6	522.548	0.669	6	14.9	140.731	15	3.529	-2.866	8.781	43.774	Sugar
Cupressoside A	934371-82-1	492.522	1.249	5	13.2	141.015	12	3.796	-4.331	9.111	46.642	Sugar
Podophyllotoxin glucoside (6CI,7CI)	16481-54-2	576.553	0.415	4	16.95	142.639	10	7.577	-1.606	8.963	42.944	Sugar
Secoisolariciresinol diglucoside	158932-33-3	686.706	-1.686	10	23.4	203.682	25	3.628	-0.446	9.085	47.438	Sugar
